# Malaria Elimination Campaigns in the Lake Kariba Region of Zambia: A Spatial Dynamical Model

**DOI:** 10.1371/journal.pcbi.1005192

**Published:** 2016-11-23

**Authors:** Milen Nikolov, Caitlin A. Bever, Alexander Upfill-Brown, Busiku Hamainza, John M. Miller, Philip A. Eckhoff, Edward A. Wenger, Jaline Gerardin

**Affiliations:** 1 Institute for Disease Modeling, Bellevue, WA, United States; 2 National Malaria Control Centre, Lusaka, Zambia; 3 PATH Malaria Control and Elimination Program in Africa (MACEPA), Lusaka, Zambia; University of California, Los Angeles, UNITED STATES

## Abstract

As more regions approach malaria elimination, understanding how different interventions interact to reduce transmission becomes critical. The Lake Kariba area of Southern Province, Zambia, is part of a multi-country elimination effort and presents a particular challenge as it is an interconnected region of variable transmission intensities. In 2012–13, six rounds of mass test-and-treat drug campaigns were carried out in the Lake Kariba region. A spatial dynamical model of malaria transmission in the Lake Kariba area, with transmission and climate modeled at the village scale, was calibrated to the 2012–13 prevalence survey data, with case management rates, insecticide-treated net usage, and drug campaign coverage informed by surveillance. The model captured the spatio-temporal trends of decline and rebound in malaria prevalence in 2012–13 at the village scale. Various interventions implemented between 2016–22 were simulated to compare their effects on reducing regional transmission and achieving and maintaining elimination through 2030. Simulations predict that elimination requires sustaining high coverage with vector control over several years. When vector control measures are well-implemented, targeted mass drug campaigns in high-burden areas further increase the likelihood of elimination, although drug campaigns cannot compensate for insufficient vector control. If infections are regularly imported from outside the region into highly receptive areas, vector control must be maintained within the region until importations cease. Elimination in the Lake Kariba region is possible, although human movement both within and from outside the region risk damaging the success of elimination programs.

## Author Summary

What would it take to eliminate malaria in southern Africa? While the past century has seen many countries eliminate malaria, and many regions have dramatically reduced malaria burden in the last fifteen years, no sub-Saharan African country has yet eliminated malaria. In the Lake Kariba region of Southern Province, Zambia, villages with high and low malaria burden are interconnected through human travel, making elimination potentially very challenging. We used detailed spatial surveillance data—including household locations, climate, clinical malaria incidence, prevalence of malaria infections, and bednet usage rates—to construct a model of interconnected villages in the Lake Kariba region, then tested a variety of intervention scenarios to see which ones could lead to elimination. We found that elimination is indeed possible and requires high, yet realistic, levels of bednet usage among area residents. Mass drug campaigns deployed to kill parasites within the human population can boost the chances of achieving elimination as long as bednet usage is already high.

## Introduction

Malaria is a vector-borne parasitic disease affecting millions of people worldwide, with *Plasmodium falciparum* still causing over 400,000 deaths per year [1]. Recent escalation in vector control has greatly reduced global burden and brought many regions close to elimination [2]. In some settings, mass drug campaigns have been an effective tool for depleting the human infectious reservoir and breaking the cycle of transmission, although the effectiveness of such campaigns has been mixed [3].

The ultimate goal is global malaria eradication, but the extreme heterogeneity in transmission levels, vector bionomics, health systems, and population densities limit the applicability of any single strategy [4]. Elimination at a single country level could be challenging to maintain in the presence of high cross-border movements of infected individuals, depending on the country and its regional context [4]. As a result, the concept and implementation of regional malaria eliminations provides a useful staging for progress towards eventual global eradication [5].

Southern Africa is one region where programs are planning operational strategies for elimination [6]. Elimination in southern Africa requires elimination in the Lake Kariba region of Southern Province, Zambia, where areas of high- and low-intensity transmission are interconnected. Understanding how to achieve elimination in the microcosm of the Lake Kariba area would provide a solution to how to achieve elimination in Southern Africa and possibly in a number of other challenging settings.

**Fig 1:**
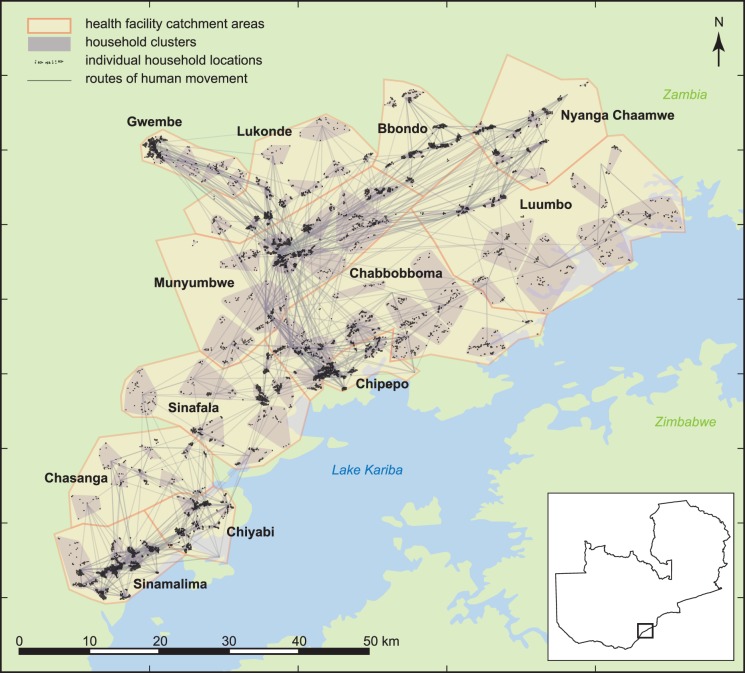
Households in the Lake Kariba region of Southern Province, Zambia, are clustered into village-scale simulation constructs within twelve health facility catchment areas. Adapted from [9].

The Zambia National Malaria Control Centre has successfully scaled up recommended malaria control interventions over the past decade and is pursuing alternative methods to further reduce malaria transmission, including community-targeted parasite reservoir reduction strategies [7,8], with a target elimination date of 2020. Beginning in 2012, mass drug campaigns have been carried out annually in the Lake Kariba region of Southern Province, Zambia (Fig 1), where transmission is seasonal and spatially variable.

Understanding the small-scale variation in the interconnected Lake Kariba region through mathematical modeling provides important insights into the critical thresholds for successful outcomes. Previous modeling efforts have provided guidance for conducting successful campaigns in generic settings [10–12], yet limited work has been done on understanding how a specific geography's individual features can also affect campaign outcomes [13,14] or how spatial variation in vectorial capacity can sustain transmission in interconnected areas. In this work, the most detailed spatial model of a specific geography to date is constructed and used to predict how various factors such as variation in transmission intensity and human migration patterns interact to influence the success of control and elimination efforts.

Local transmission dynamics were reconstructed within a mechanistic model of malaria transmission using the high-resolution surveillance data collected during the mass test-and-treat (MTAT) campaigns of 2012–13 in the Lake Kariba region, which included infection status, age, GPS coordinates of households, recent symptoms, recent treatment, and insecticide-treated net (ITN) usage. Village-scale biting rates were selected to calibrate local malaria prevalence and incidence to longitudinal surveillance data and seasonal patterns of clinical case counts reported at health facilities respectively.

The simulation framework was then used to assess the outcome of a variety of post-2016 intervention scenarios. Simulations predict that high coverage with vector control is a necessary condition of achieving elimination in this region, and mass drug administrations (MDAs) are effective at increasing the likelihood of elimination only if excellent vector control is already in place. Importations of infections into the Lake Kariba area from outside the region present a particular challenge to maintaining elimination if the importations occur in highly receptive areas, and vector control should be sustained in those areas to prevent outbreaks and reestablishment of endemic malaria as long as importations continue.

## Results

### Malaria transmission in the Lake Kariba region

A high-resolution spatial model of twelve health facility catchment areas (HFCAs) in the Lake Kariba region was configured based on village-scale clusters of households and ITN usage, MTAT coverage, case management, and human migration rates derived from the surveys conducted in 2012–13 (Fig 1 and S1–S7 Figs, Methods). Malaria transmission was modeled within each cluster, where vector populations were driven by cluster-specific climate data and local abundance of larval habitats.

**Fig 2:**
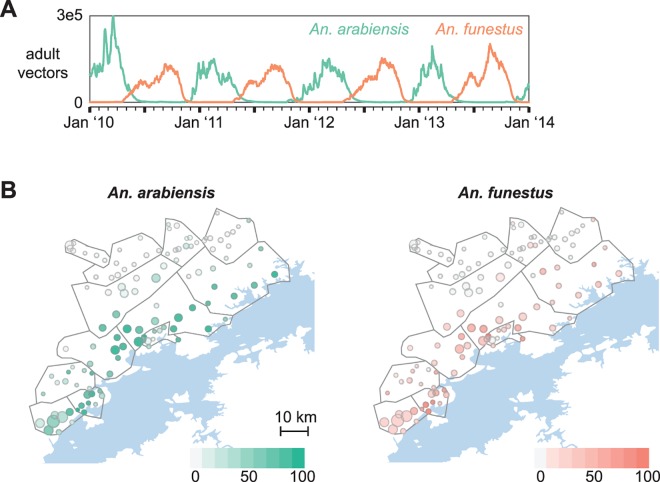
Spatial distribution of two vector species governs magnitude and seasonality of malaria transmission over the Lake Kariba region. (A) Adult vector numbers are tuned by scaling larval habitat availability of *An*. *arabiensis* and *An*. *funestus* vectors. Relative scales of the two vector species govern the seasonality of human biting. Shown: *arabiensis* scale = 100, *funestus* scale = 30. (B) Best fit *arabiensis* and *funestus* larval habitat availabilities vary spatially over the study region, with more habitat available for both species in the lower-altitude regions closer to the lake front.

**Fig 3:**
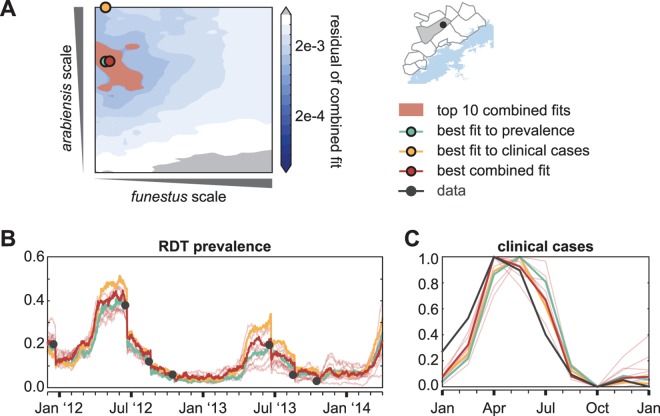
Representative calibration of larval habitat availabilities in a single cluster located in Munyumbwe HFCA. (A) Sampling over larval habitat availability scale factors for *arabiensis* and *funestus* identifies pairs of scale factors with good fits to: cluster-level prevalence by rapid diagnostic test (RDT) from surveillance data (blue, best fit to prevalence), HFCA-level seasonality of weekly reported clinical cases counts (yellow, best fit to clinical cases), and both prevalence and seasonality of clinical cases (red, best combined fit). Surface shows the residual error in the combined fit to both prevalence and clinical cases. (B) RDT prevalence between December 2011 and April 2014 observed during surveillance (black) and in simulation. Blue: Simulation of single pair of *arabiensis* and *funestus* habitat availability scales that result in the best fit to the surveilled prevalence without accounting for fit to seasonality of clinical case counts. Yellow: Simulation of single pair of habitat scales that result in the best fit to seasonality of clinical case counts without accounting for fit to prevalence. Red: Simulation of pairs of habitat scales that result in best (bold line) and top 10 (thin lines) fits when optimizing fit to both surveilled prevalence and seasonality of reported clinical case counts. (C) Normalized seasonality of clinical case counts observed in Munyumbwe health clinic reported data (black) and simulation of a single Munyumbwe cluster (blue, yellow, red, defined as in panel B).

Preliminary entomological data indicated that both *Anopheles arabiensis* and *Anopheles funestus* are present in the study area (personal communication with Javan Chanda), with *arabiensis* biting rates highest between January and April during the warm rainy season while *funestus* peaks in September at the beginning of the hot dry season (Fig 2A). Relative abundances of *arabiensis* and *funestus* govern the seasonality of malaria transmission, while absolute abundances determine the intensity. For each village-scale cluster, combinations of larval habitat availabilities for *arabiensis* and *funestus* were simulated with appropriate patterns of ITN usage, case management rates, and MTAT coverage. The resulting simulated prevalence and clinical case counts were compared with surveillance data to select the combination of habitats yielding the best fit to field observations (Fig 3 and S8 Fig, Methods).

Calibration to surveillance data resulted in the expected gradient of higher habitat availability of both vector species closer to Lake Kariba and lower availability in the higher-altitude HFCAs more distant from the lake (Fig 2B). Chiyabi HFCA was predicted to have the largest amount of *funestus* in this region, suggesting that ITNs may be particularly effective there as *funestus* is indoor-feeding and highly anthropophilic [15].

**Fig 4:**
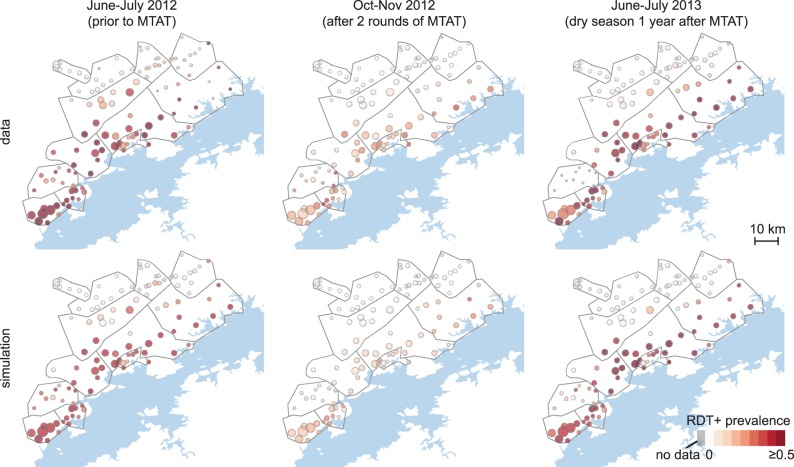
Simulations capture spatio-temporal variation in cluster-level prevalence of RDT-positive infections. Top: survey data collected during MTAT rounds. Bottom: mean cluster prevalence in 100 samples from the joint posterior distribution of 10 best-fit habitat availability pairs for each cluster.

The calibrated simulations captured both fine-scale cluster-level variation in malaria prevalence and large-scale spatio-temporal trends of temporary reduction in prevalence observed in the study area following MTAT rounds (Fig 4 and S9 Fig). Surveillance in 2012–13 reported stalled reduction in prevalence by late 2012 and rapid rebound after the following rainy season. This observation was replicated in the spatial model, where significant re-infection between rounds two and three in lakeside clusters drives rebound throughout the study area. The model was able to predict cluster-level prevalence in December 2014 with reasonable accuracy (S10 Fig, r^2^ = 0.46) considering that local variation in 2014 vector control implementation, location-specific migration patterns, and location-specific changes in health-seeking behavior as a result of the community health worker and case investigation programs that began in mid-2014 were not part of our simplified forward-simulation scenarios.

**Fig 5:**
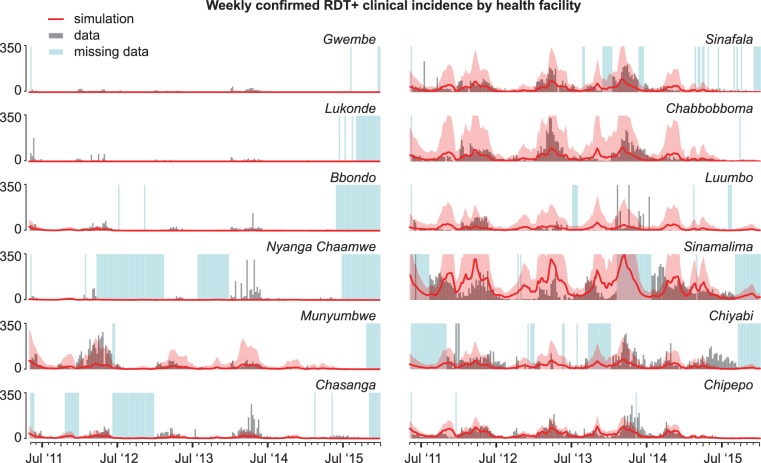
Simulations capture the seasonality of HFCA-level weekly clinical case counts. Simulated clinical and severe malaria cases are scaled by sampling treatment-seeking rates of clusters whose distance to their health facilities are within 2km of the target cluster’s distance to its health facility. Simulated case counts were also scaled by 150% in all HFCAs to account for underestimating catchment populations due to incomplete coverage during MTAT. Mean (line) and range (shaded area) of 100 samples from the joint posterior distribution of 10 best-fit habitat availability pairs for each cluster.

The observed seasonality of weekly clinical cases confirmed by rapid diagnostic test (RDT) was well-captured by simulations, including a characteristic pattern of high case counts between December and June and a small rise in cases in Chipepo and Sinamalima HFCAs as temperatures rise in October (Fig 5). Seven HFCAs had strong Spearman’s rank correlation between simulation and observed clinical cases (rank correlation values between 0.62 and 0.83), Luumbo and Sinamalima HFCAs had moderate correlation (values 0.45 and 0.51), Chiyabi HFCA had weak correlation (0.23), Gwembe HFCA had very weak correlation (-0.03), and Nyanga Chaamwe HFCA had a weak negative correlation (-0.27) likely due to inadequate data. With the exception of Gwembe HFCA, model fits to clinical cases were better for HFCAs with consistent reporting. Individual clusters also occasionally saw discrepancies between observed seasonality of clinical cases at the local health facility and simulated seasonality at the cluster (Fig 3C). On the modeling side, these differences could be due to limited knowledge of how local climate drives mosquito abundances, including the rates at which temporary larval habitats appear and disappear, or how infection, clinical symptoms, and health-seeking are related in this region.

Many factors drive uncertainty in both magnitude and seasonality of observed case counts. While simulations estimate a constant proportion of clinical cases seek care at health facilities, several factors could contribute to discrepancies between true and observed clinical incidence in the field. Gwembe HFCA, which otherwise has very low transmission, contains the district hospital. Clinical cases reported from Gwembe HFCA may have traveled from elsewhere within or outside the study area expressly to seek care. Case management rates in simulation were estimated from survey responses during the dry season, while individuals with fever during the wet season may show different health-seeking behavior based on road conditions and personal assessment of whether the fever is malarial. A strong distance-dependence on health-seeking means households closer to the health facility contribute disproportionately more to recorded case counts than to true clinical incidence, yet this data shows significant variation (S4B Fig). Clinics report any RDT-positive individual presenting with fever as a clinical case of malaria. However, a substantial portion of individuals in malaria-endemic areas with non-malarial fevers will be RDT-positive due to low-density infection or recent clearance of malaria [16,17]. Finally, the population denominator in this area is unknown, and preliminary analysis from cross-referencing individuals across all surveillance rounds suggests that the true population may be nearly twice as high as the simulation population, which was chosen based on estimates from operations teams working in the region.

### Regional elimination requires high coverage with ITNs

Incomplete coverage and imperfect diagnostics in MTAT campaigns result in a significant portion of the parasite reservoir being left untreated [18–21]. This untreated reservoir will resume the cycle of transmission during the next rainy season. However, a mass-distribution mode that can treat undetected infections and a drug formulation such as dihydroartemisinin-piperaquine (DP), which has longer prophylactic protection against re-infection than artemether-lumefantrine (AL), the drug used in the MTATs, may be more successful at interrupting transmission [11].

Operations teams in the Lake Kariba region continued mass treatment following the 2012–13 MTAT by administering MDA and focal MDA to a randomized group of HFCAs. Simulations approximated operational activities in 2014–15 by administering a mass distribution of ITNs in June 2014 and MDA with DP to all HFCAs in December 2014 and July 2015, in line with operational schedules.

**Fig 6:**
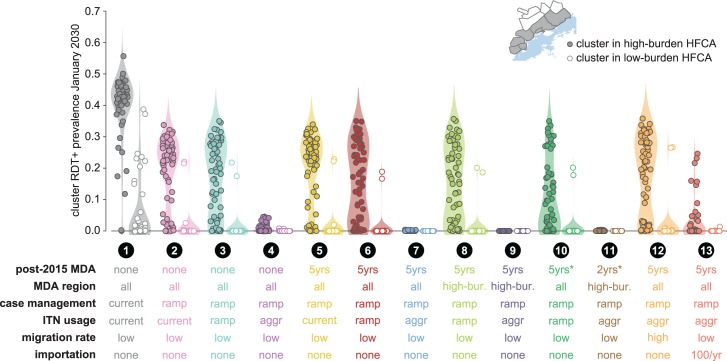
Prevalence of RDT+ infections in the Lake Kariba region a decade after target elimination date of 2020 under various post-2015 intervention scenarios. Clusters are divided into high-burden (n = 64) and low-burden (n = 51) groups as indicated in the inset map. Cluster prevalence in each scenario is the mean of 100 samples from the joint posterior distribution of 10 best-fit habitat availability pairs for each cluster. Post-2015 MDA: MDA is distributed in 2014 and 2015 to all HFCAs in all scenarios. Scenarios 1–4: MDAs discontinued after 2015. Scenarios 5–10, 12–13: MDAs continue annually between 2016 and 2020, a total of 5 additional distributions. Scenario 11: MDA is distributed only in 2017 and 2018. Scenarios 10–11, indicated with asterisk: post-2015 MDAs have 70% coverage. All other MDA distributions have coverage as indicated in S5 Fig. MDA region: MDAs are distributed to clusters in all HFCAs except in scenarios 8, 9, and 11, where only clusters in high-burden HFCAs receive MDAs after 2015. Case management: In scenario 1, case management is maintained at rates observed during 2012–13 surveillance. In all other scenarios, case management rates increase and plateau as shown in S4 Fig. ITN usage: In scenarios 1, 2, and 5 (“current”), ITNs are not distributed after 2015. In scenarios 3, 6, 8, and 10 (“ramp”), ITN usage after 2015 ramps up following historical rates. In all other scenarios (“aggr”), ITNs are distributed at an aggressive 80% coverage biannually between 2018–22. Importation: Infections imported from outside the 12-HFCA study area.

**Fig 7:**
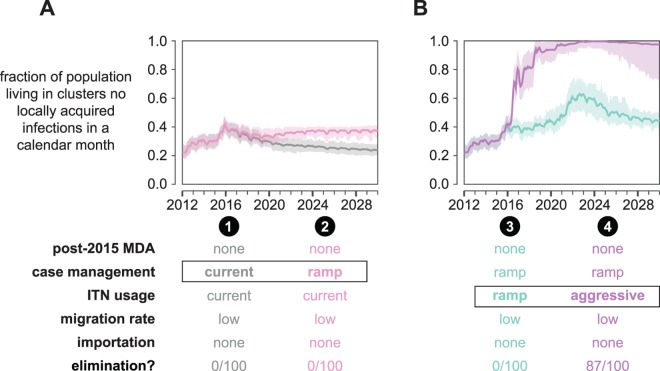
Malaria elimination in the Lake Kariba region is possible under high levels of ITN usage even without distributing MDAs after 2015. The fraction of total study area population living in clusters where no local transmission has occurred over a month-long period is plotted for each month between January 2012 and January 2030. Line indicates the mean and shaded area the range observed over 100 samples from the joint posterior distribution of 10 best-fit habitat availability pairs for each cluster. A simulation results in elimination if no new infections occur in all clusters over a 3-year period. The “elimination” row indicates the fraction of simulations where elimination was observed. (A) If ITN distributions stop after 2015, elimination is never observed to occur. (B) Under an aggressive ITN distribution scenario, elimination becomes likely even without additional MDAs after 2015.

**Fig 8:**
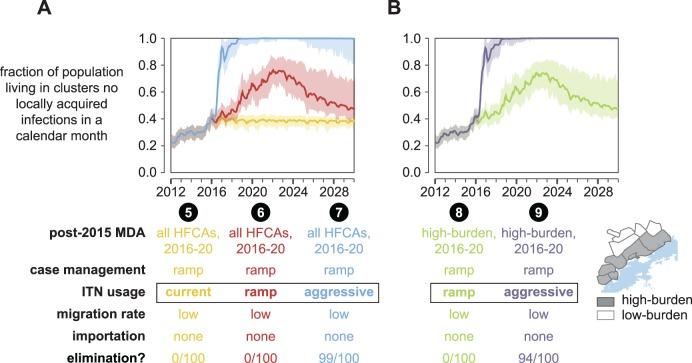
Administering five more years of MDA to all or a subset of HFCAs increases the likelihood of elimination under high levels of ITN usage but cannot achieve elimination on its own. The fraction of total study area population living in clusters where no local transmission has occurred over a month-long period is plotted for each month between January 2012 and January 2030. Line indicates the mean and shaded area the range observed over 100 samples from the joint posterior distribution of 10 best-fit habitat availability pairs for each cluster. A simulation results in elimination if no new infections occur in all clusters over a 3-year period. The “elimination” row indicates the fraction of simulations where elimination was observed. (A) Five more years of MDA in all HFCAs increases the likelihood of elimination only when ITN usage is very high. Otherwise, elimination remains very unlikely. (B) Limiting the 2016–20 MDAs to high-burden areas yields similar results to simulations where MDA was distributed to all clusters.

**Fig 9:**
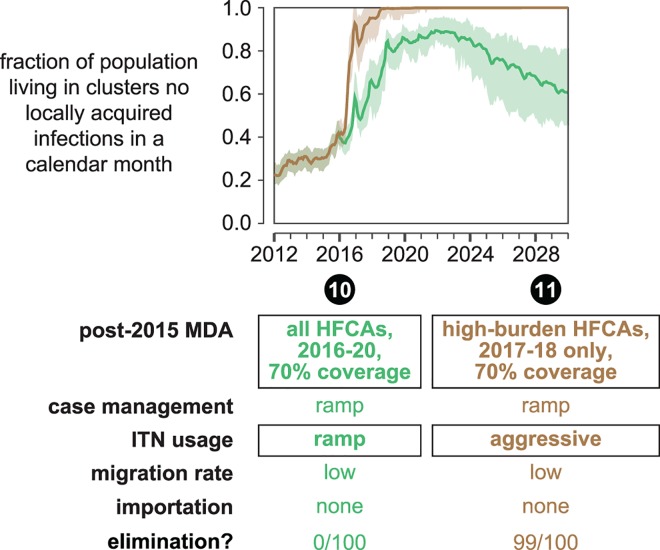
Higher MDA coverage cannot overcome insufficient vector control. In scenarios 10 and 11, post-2015 MDAs have a higher coverage of 70%. The fraction of total study area population living in clusters where no local transmission has occurred over a month-long period is plotted for each month between January 2012 and January 2030. Line indicates the mean and shaded area the range observed over 100 samples from the joint posterior distribution of 10 best-fit habitat availability pairs for each cluster. A simulation results in elimination if no new infections occur in all clusters over a 3-year period. The “elimination” row indicates the fraction of simulations where elimination was observed.

**Fig 10:**
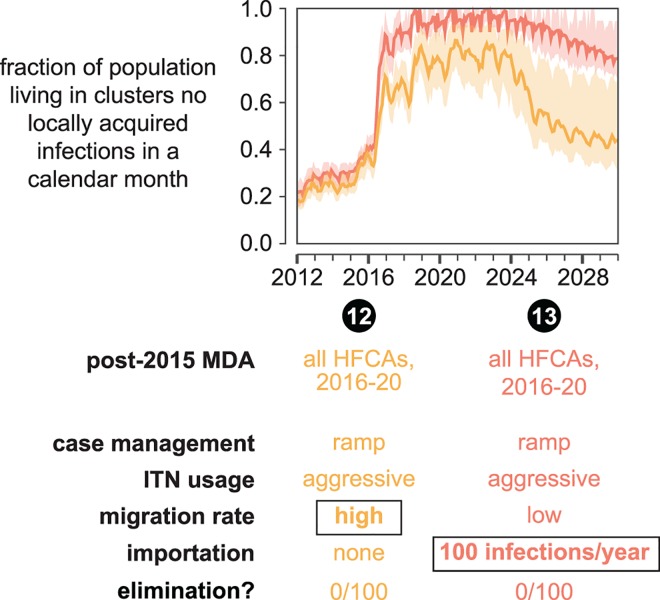
Human movement can disrupt the success of an elimination program. Both increased movement within the 12 HFCAs as well as importation of infections into the region can drastically decrease the likelihood of elimination in the Lake Kariba region. The fraction of total study area population living in clusters where no local transmission has occurred over a month-long period is plotted for each month between January 2012 and January 2030. Line indicates the mean and shaded area the range observed over 100 samples from the joint posterior distribution of 10 best-fit habitat availability pairs for each cluster. A simulation results in elimination if no new infections occur in all clusters over a 3-year period. The “elimination” row indicates the fraction of simulations where elimination was observed.

To compare how case management, ITN usage, and MDA coverage contribute to reducing malaria burden separately and together, a variety of post-2015 intervention scenarios were simulated (Figs 6–10). For each simulation, the fraction of total study area population living in clusters where no local transmission has occurred over a month-long period was measured for each month between January 2012 and January 2030. Elimination was counted to have been achieved if no infection events occurred anywhere in the simulated study area over a continuous three-year period.

As expected, clusters in high-burden HFCAs were more likely to retain transmission in all non-eliminating scenarios (Fig 6). Increasing passive case management rates without also distributing more ITNs or implementing any MDA campaigns resulted in nearly all low-burden clusters maintaining very low prevalence through January 2030 and likely interrupting local transmission in many low-burden HFCAs (scenario 2). Elimination within the Lake Kariba region thus becomes a question of which interventions are effective at reducing transmission in the high-burden clusters.

Discontinuing MDAs after the 2015 rounds while maintaining current levels of ITN usage did not result in long-term reduction in regional malaria transmission (Fig 7A, scenario 1). Increasing passive case management rates (scenario 2) established a new baseline of lower transmission during the decades following the end of MDAs in 2015 but could not achieve region-wide elimination. Combining the 2014–15 MDAs with ramp-ups in case management and increased ITN usage resulted in elimination under highly aggressive ITN distribution campaigns but not if ITN ramp-ups followed historical rates of increase (Fig 7B and S11 Fig). Under the ramp-up scenario (scenario 3), transmission rebounded after the last ITN distribution in 2022 to a new baseline largely determined by case management rate. In the aggressive scenario (scenario 4), 87% of simulations resulted in elimination.

The aggressive ITN distribution schedule consists of mass distributions with 80% coverage every two years between 2016 and 2022. As a result, 75–90% of the entire study area population is sleeping under an effective net every night over a 9-year period (S11F Fig). Under the ramp-up schedule, ITN usage with effective nets peaks at only 50–80%. This difference in maximum coverage achieved is enough to prevent elimination from occurring in the simulated scenarios.

Can MDA compensate for insufficient vector control? Extending annual dry-season MDAs through 2020 increased the probability of elimination under the aggressive ITN distribution schedule from 0.87 to 0.99 (Fig 8A, scenario 7), but if ITN usage was maintained at current levels or ramped up gradually, elimination was still never observed (scenarios 5 and 6).

Restricting post-2015 MDAs to high-burden HFCAs resulted in outcomes comparable to cases where MDAs were distributed to all HFCAs, with no elimination observed under the ramped ITN distribution and probability of elimination falling slightly from 0.99 to 0.94 under the aggressive ITN distribution (Fig 8B, scenarios 8 and 9), although the boosting effect of MDA on top of the aggressive ITN distribution campaigns was not as large as when MDA was implemented in all HFCAs: adding MDA to high-burden areas only increased probability of elimination from 0.87 to 0.94 while MDA in all areas increased probability of elimination from 0.87 to 0.99. In the small fraction of cases where the combination of aggressive ITN distributions and MDAs did not result in elimination, residual transmission remaining in the densely populated Sinamalima and Chiyabi HFCAs was able to reestablish transmission more broadly in the study area (S12 Fig).

In all scenarios, MDA coverage is simulated at levels observed during the 2012–13 MTAT campaigns. It is possible that MDA coverage could increase in later rounds as campaign logistics are improved. Scenario 10 in Fig 9 shows results of higher-coverage MDAs given on top of the ramped ITN distributions, similar to scenario 6 but with MDA coverage increased to 70%. Elimination is never observed in these simulations. In contrast, even two years of MDA at 70% coverage in high-burden clusters (Fig 9, scenario 11) boosts probability of elimination from 0.87 to 0.99 if high coverage vector control is already present.

Human movement within the Lake Kariba region connects areas of high and low transmission. In scenario 12 (Fig 10), migration rates were increased ten-fold from the base level used in all other scenario projections. Under these conditions, intervention packages that previously resulted in elimination (scenario 7) now have much smaller effects on transmission within the region: elimination is never observed. While the migration rate in scenario 12 is likely to be much higher than the true migration rate, this scenario demonstrates that outcome of an elimination program is highly dependent on the nature and extent of regional human movement. While efforts are beginning to be made to characterize relevant human migration patterns [22–24], understanding local geographies and conditions will ultimately be crucial for predicting whether an elimination program will succeed.

None of scenarios 1–12 include importation from outside the modeled region. While the Lake Kariba districts as a whole represent an isolated area of higher prevalence surrounded by lower-transmission areas [2], it is possible that travelers from further afield may bring infections into the area. In scenario 13, a total of 100 infections are imported twice a year into three highly connected, high-population clusters in Gwembe, Munyumbwe, and Sinamalima HFCAs. This importation rate is likely to be higher than the true importation rate, especially during later years of simulation if prevalence follows recent trends and continues to decline throughout southern Africa, and thus represents an upper bound on the effect of imported infections. Including these importations in a simulation with the same intervention mix as the highly successful scenario 7 resulted in no simulations achieving elimination. Maintaining routine vector control operations such as annual spraying with a long-lasting insecticide is sufficient to prevent widespread resurgence, although elimination remains unlikely as long as importations continue (S13 Fig). Elimination programs for a given area should not be designed in isolation but rather in the context of the broader region, as importation pressure from outside the elimination area can potentially disrupt desired outcomes inside the area.

## Discussion

The complex spatial dynamics of malaria transmission in the Lake Kariba region of Southern Zambia were captured in a mathematical model, which was then extended to compare the effects of future interventions. The simulation framework was informed by household surveillance in 2012–13, clinical case counts reported by local clinics, and preliminary entomological data. This work is the first to examine how village-scale variation in transmission intensity can drive transmission throughout an interconnected region.

While the 2012–13 MTAT surveys were a rich source of data for model-building, they presented an incomplete picture of local malaria transmission because measurements were taken during the dry season. The seasonality of transmission was thus difficult to characterize based solely on these measurements. Weekly clinical case counts reported from health facilities were broadly informative of transmission during the remaining months of the year, but ascertaining the proportion of RDT-positive fevers that are true malarial fevers is difficult. The seasonality of cases observed at the health facility may not reflect seasonality of transmission in every village within the HFCA as each HFCA may encompass a range of transmission intensities. Nonetheless, simulations were able to recapture both region- and village-scale spatio-temporal features of transmission observed during the 2012–13 operations.

Local entomology drives the seasonality of transmission. Attempting to model the study area with only *arabiensis* vectors requires non-seasonal year-round transmission in many lakeside areas in order to capture substantial prevalence during the November surveys, which would result in a mismatch with observed seasonality of clinical case counts. Incorporating *funestus* allowed the model to capture both dry season prevalence and seasonal patterns of clinical cases. More entomological data on vector species abundance and behavior will guide continued model refinements.

All simulations assumed full compliance with drug regimens. While perfect adherence to treatment is unlikely [25,26], elimination is possible even when compliance is extremely poor (S14 Fig), and previous modeling work has shown that compliance is not crucial in mass campaigns in settings without drug resistance [11]. Most individuals who harbor dry season infections have low parasite densities [16,18], and a single dose of antimalarial drug is often sufficient to clear those infections.

Excellent vector control is the single most important intervention for achieving elimination. Without sustained high coverage with ITNs, elimination is never observed in any of the scenarios tested, and when ITN usage is maintained at high levels over many years, elimination is observed in 87% of simulation runs. In this study, vector control has been modeled exclusively as ITN usage, but indoor residual spraying (IRS) would have similar effects if local vectors are susceptible. Combining IRS and ITNs likely represents a more attainable scenario for aggressive vector control given that ITN usage has an unpredictable behavioral component while IRS remains in place unless walls are washed or re-plastered. The simulations of the Lake Kariba area presented here are dominated by *arabiensis* vectors, which have been modeled with a 50% indoor biting rate. Regions where indoor biting predominates may require slightly less intense vector control interventions to achieve the same elimination outcomes.

Unless vector control is also sustained at high coverage, implementing MDA campaigns will not result in elimination and is not a good use of valuable resources. High coverage in drug campaigns is difficult to achieve, and simulations predicted that drug campaigns with coverage as high as 70% could not compensate for insufficient underlying vector control. As MDAs are expensive to administer, we encourage elimination programs to consider whether their operational region meets the preconditions we have discussed here for effective MDA before deciding to implement this intervention.

Simulations predict that if MDAs are implemented on top of a very aggressive vector control program, elimination becomes even more likely. As predicted by generic models [27], however, even in this context transmission can occasionally rebound after vector control ceases (Fig 8 and S12 Fig). Rebound in transmission depends on the extent of human movement as travelers from high-transmission areas re-seed transmission in low-transmission areas. If vector control is well-implemented at high coverage, targeting MDAs to high-burden areas is potentially a reasonable approach to increasing the likelihood of local elimination while saving resources, and could even result in improved outcomes if better coverage with MDA can be achieved in targeted areas.

As long as passive case management improves, simulations predict that malaria transmission will die out in low-burden areas. Human movement inside the study area and importation of infection from outside the study area decrease the likelihood of elimination. If certain areas are known to be hot spots of both importation and receptivity, vector control should be implemented as a preventative measure until elimination is achieved in the wider region and imported infections cease. Surveillance must be vigilant in quickly identifying local outbreaks and responding with high-coverage vector control and possibly focal MDA around index cases. If outbreaks are not adequately contained and endemic malaria is reestablished, sustained high coverage with vector control must be re-implemented and MDA should be considered to rapidly deplete the parasite reservoir before other areas also experience resurgence. Elimination in the face of repeated importations is a major challenge and victory should not be declared too soon, as relaxing surveillance prematurely can easily lead to resurgence in areas with a history of high transmission.

While generic models have predicted that targeting transmission foci would be effective [28], this study demonstrates for the first time that elimination strategies in interconnected regions are well-served by focusing directly on reducing transmission in regional hotspots rather than attempting to maintain an “elimination front” and tackling the most difficult areas last. Programs will need to identify where regional hotspots are located, with the understanding that hotspot locations and intensities can change dynamically as interventions are deployed and changes in vector species composition, human movement patterns, housing conditions, and climatic cycles alter the local landscape of transmission.

As more regions reduce transmission and approach malaria elimination, it becomes crucial to understand how to set up malaria operations for successful and lasting elimination at local, national, and regional levels. Southern Africa is a particular challenge as vectorial capacity is substantial in some areas and the region has become increasingly interconnected. This study suggests that elimination in the southern Africa region will require several years of sustained high coverage with vector control interventions, and mass drug campaigns are unlikely to be effective if vector control is insufficient. In areas with both high receptivity and high importation rates, vector control must be maintained until importation ceases. Ultimately, it follows that regional malaria elimination in southern Africa is nevertheless within reach with current tools, provided the efficacy and operational efficiency attained in the Lake Kariba operational area can be extended and targeted to other key areas.

## Methods

### Study site overview

During each of the 2012 and 2013 dry seasons (June-November) in Southern Province, Zambia, three large-scale MTAT rounds were undertaken [7]. Individuals were visited at their homes in a full community census and, following consent, administered RDTs. Test-positive individuals were treated with the antimalarial drug artemether-lumefantrine (AL), of which the first dose was directly observed. During each MTAT round, RDT results were geo-tagged by household location. Information was collected on household demographics, ITN usage, recent fevers, and recent drug treatments.

In this analysis, focus was restricted to a contiguous block of twelve HFCAs in Gwembe and Sinazongwe Districts along Lake Kariba (Fig 1). These HFCAs cover approximately 80,000 people living in a geographic area spanning a range of endemic malaria transmission intensities. Demographics, migration, ITN usage, treatment-seeking, and drug campaign coverage in simulations were informed by survey data collected during the MTAT rounds. Spatial variation in transmission intensity was captured by selecting local vector larval habitat availabilities to match observed seasonal and spatial patterns in RDT prevalence and clinical incidence.

### Constructing a detailed spatial model of malaria transmission in the Lake Kariba region

#### EMOD model of malaria transmission

High-resolution spatial simulations were conducted with EMOD v2.0, a mechanistic, individual-based model of malaria transmission with temperature-dependent vector life cycle dynamics [29] and acquired human immunity to asexual parasites [30–33]. Asexual and sexual stage parasite densities are explicitly modeled within each infected individual. Age-dependence of prevalence, incidence, parasite densities, and infection durations have been calibrated to field data from a variety of African study sites as well as malariatherapy data [18,34].

Within EMOD, RDTs test for the presence of asexual parasites in an individual’s blood. A parasite count is drawn from a Poisson distribution around the true parasite count and compared with the diagnostic’s sensitivity. All RDTs administered in simulation had sensitivity of 40 asexual parasites/μL.

#### Population demographics

Household locations within the 12 HFCAs were aggregated to 115 village-sized clusters using a *k-means* algorithm, restricting clusters to reside within HFCAs (Fig 1). The number of clusters in each HFCA, *k¸* was selected such that increasing *k* by one yielded a less than 10% improvement in within-cluster sum of squares. The population in each simulation cluster averaged a few hundred people (S1 Fig) and varied from round to round due to inter-cluster migration and cycling from births and deaths. Birth and death rates were fixed at 45 per thousand per year in all simulations.

Migration rates between clusters were approximated using a truncated gravity model based on estimated pairwise travel times. First, an adjacency graph was constructed with distance-weighted edges connecting all pairs of clusters within 8.5 km, representative of foot paths and minor roads, and with lower travel-time-normalized distance weights on edges connecting nodes along several known paved roads, for instance D375 and D500 in Gwembe (Fig 1). Next, the shortest travel times on this adjacency graph were calculated using the Dijkstra algorithm between all pairs of clusters. Finally, the migration rates were estimated using a gravity model of the form

$$M=G\frac{P}{d^{2}}$$ (1)

where *P* is the destination population, *d* is the travel-time-normalized distance, and *G* is a scale parameter. The value of scale parameter *G* was explored over a broad range that covers expectations from population surveys [22]. A minimum distance between clusters of 1 km was imposed to improve short-distance asymptotic behavior. Migration events were not modeled in an age or seasonally dependent fashion, and 95% of all trips were round trips with duration drawn from an exponential distribution with a mean of two days.

#### Spatial climate data model

Daily mean air temperature and humidity were estimated for the period from 2006–2013 with a climate algorithm applied to GSOD weather-station data (S2 Fig) [35]. Rainfall data were obtained from the NOAA African Rainfall Estimation Algorithm [36]. Cluster-specific climate data modulated daily vector populations and larval habitat availability year-round, to account for seasonal, topological and environmental effects.

#### ITN coverage and efficacy

Personal and community effects of ITN usage vary within the model according to parameterizations of usage patterns, killing and blocking utility, and vector species behavior.

The fraction of the population using ITNs in each cluster of the study site in 2012–13 was estimated using cluster-level aggregate data (S3A Fig). Historical estimates from the Malaria Atlas Project [2,37] were used to model the effect of ITN usage in the years leading to 2012–13. Different areas in the Lake Kariba region received varying amounts of ITNs between 2006 and 2011. Starting from approximately 10% coverage in 2006, some areas reached as much as 65–70% and as little as 25% ITN usage in 2011. These trends were summarized in four distinct ITN usage ramp-up trajectories (S3B Fig), consisting of four tri-annual ITN distribution campaigns. Each cluster was assigned to the ITN ramp-up trajectory resulting in the closest least square match with ITN usage rates observed in the 2012–13 surveillance data. Age-specific ITN usage was modeled according to the pattern observed in the survey data (S3C Fig), with usage among children aged 5 to 20 assumed to be half that of individuals of other ages. In addition to the age-targeted mass distribution campaigns, ITNs were also issued to individuals at birth in simulation in order to maintain the observed age pattern in ITN usage. In simulation, all individuals with nets use them every night: “usage” and “coverage” are interchangeable terms when referring to simulations. The ITN initial killing and blocking rates were set to 0.9 and 0.6 with respective half-lives of approximately 1.5 and 2.5 years, with exponential decay in ITN efficacy [37]. Community-level ITN effects were similar between surveillance and simulation data (S3D Fig).

The effectiveness of nets is strongly coupled to the feeding behavior of the dominant *Anopheles* species in the area. According to preliminary entomological data from the study site, *An*. *funestus* and *An*. *arabiensis* constitute the primary vectors. While *funestus* behavior is pronouncedly endophilic characterized by peak biting during the night and indoor resting [38], *arabiensis* feeding and resting patterns are diverse, with earlier evening biting peak and varied exophilic/endophilic behavior [39]. In the absence of detailed entomological information from the study site–especially as studies in Macha [40] and Luangwa [38] give somewhat divergent pictures–the indoor feeding fraction and ITN killing parameters were tuned to the observed protection observed in a statistical model of ITN protective effects after controlling for location, age, and season. Correspondingly, the model’s indoor feeding fraction was set to 0.95 and 0.5 for *funestus* and *arabiensis* respectively.

#### Case management

Case management was modeled uniformly across all clusters beginning in January 2007 according to the mean fraction of individuals with fever within the last two weeks who sought treatment as reported in the surveillance data (S4 Fig).

Simulations handled case management as follows. All clinical and severe cases receiving treatment were given an age-based dose of AL, with AL pharmacokinetics and parasite stage-specific pharmacodynamics explicitly modeled [11]. Individuals seeking care received treatment within 3 days of presenting with symptoms. All individuals were assumed to complete the full course of AL treatment. For children under 5 years of age, 32% of clinical cases and 72% of severe cases received treatment; for individuals over 5, 24% of clinical cases and 52% of severe cases received treatment. Treatment rates for severe disease were not available from surveillance and were assumed for simulation purposes to be approximately twice the rate of treatment for clinical disease.

Cluster-level case management rates were used solely for constructing the simulation confidence bands in Fig 5 and not in any calibration process or scenario projections. Data collected during the MTAT rounds found cluster-level health-seeking to vary with distance from the health facility and by round (S4B Fig). To calculate the simulated weekly clinical cases, case reporting rates for each cluster were sampled from observed health-seeking rates of clusters within 2km of the target cluster and reported cases aggregated over all clusters in an HFCA.

#### Drug campaign coverage

Coverage achieved during the 2012–13 MTAT campaigns was estimated by longitudinal linking of individuals found across multiple rounds. Longitudinal linking was performed by generating pairwise comparisons of all recorded individuals in round *i* with all those in round *i+1*. Each comparison assessed similarity of the record pair based on age, geographical location and Levenshtein distance between names. Gender was required to be identical. Both first and last names were scored, with allowance for the possibility that name order was swapped between rounds.

Selection criteria were assigned standard and restrictive thresholds (S1 Table). Records were considered a match when standard thresholds were met for all selection criteria. Linkage efficiencies were estimated under the assumption that, by tightly restricting all criteria but one, it is possible to estimate the error rate in the remaining criterion. The linkage efficiency for age, for example, was computed by applying the restrictive thresholds to name and geographical distances, and tallying the fraction of matches that are identified when the age difference meets the standard threshold. Overall linkage efficiencies were computed as the product of the efficiencies for age, name and geographical distance.

The 2012–13 MTAT campaigns varied in coverage across the 12 study area HFCAs. The average campaign coverage achieved in each HFCA was derived from the fraction of longitudinally linked individuals found within that HFCA in subsequent surveillance rounds, corrected by the estimated linkage efficiency (S5 Fig). Over the six MTAT rounds, coverage varied approximately from 30% to 75% across HFCAs, with Luumbo experiencing less than 10% coverage in the first round of the 2012 MTAT campaign due to patchy coverage (S6 Fig). To reflect the coverage heterogeneity inferred from surveillance data, the HFCAs were each assigned to a coverage level of 35% or 55%, and all clusters within an HFCA received the same campaign coverage for all drug campaigns in a simulation. Unless otherwise indicated, a cluster always received the same assigned coverage for each round of MTAT or MDA.

MTAT campaigns were distributed in simulation beginning in June of 2012 and 2013. Each campaign consisted of three rounds separated by 60 days, and all clusters received MTAT on the same day. Individuals who tested positive by RDT received AL. Coverage was independent between rounds, and full compliance with the full course of treatment was assumed. 81 clusters were part of MACEPA’s pilot study and also received a single round of MTAT in December 2011.

### Calibration of regional malaria transmission

#### Calibration sweep parameters

Given cluster-specific ITN usage and MTAT coverage, cluster-specific transmission intensity was determined by sampling over a range of larval habitat availabilities for *Anopheles arabiensis* and *Anopheles funestus*. Both cluster-level prevalence by RDT from the six MTAT rounds in 2012–13 and the December 2011 pilot round as well as weekly clinical case counts from the Zambia National Malaria Control Centre’s Malaria Rapid Report system were used to inform the selection of habitat availability at each cluster. Fit quality was evaluated based on the weighted sum of mean square error of prevalence and rank correlation of clinical incidence. During calibration, each cluster was simulated separately in a non-spatial framework.

Both year-round larval habitat sites and rainfall-driven sites–e.g. puddles replenished by rainfall and depleted by seasonal evaporation–were modeled for *arabiensis* to reflect the breeding preferences of the species. For *funestus*, larval habitat sites characterized by vegetation around the edges of water bodies were modeled with two components, one driven by rainfall to model highly transient habitats and one with a forced monthly relative availability of [0, 0, 0, 0, 0.2, 1, 1, 1, 0.5, 0.2, 0, 0] for January through December respectively to model fluctuations in lake and stream levels that are driven by longer-term cycles of water accumulation and evaporation. The monthly profile was based on preliminary entomological data from the study site on monthly *funestus* abundance as well as satellite imagery of the study area.

Given nominal capacities of year-round constant and transient water-based vegetation habitats, two model parameters were tuned–modulating respectively *arabiensis* seasonal, rainfall-driven habitats and *funestus* monthly habitat scales–over four orders of magnitude.

Since climate variation between closely geo-colocated clusters was insignificant, four reference climate locations were picked at representative altitudes and locations (S2 Fig). Each cluster *c* was assigned to the closest reference climate based on absolute altitude difference between *c* and the reference climate location. Post-assignment, malaria transmission intensities simulated with reference and actual climate data for each cluster were compared to verify that no distortion took place due to reference climate timeseries offset with respect to the actual cluster climate. All climate traces (relative humidity, temperature, and rainfall) of a reference climate category were compared to the climate traces at the actual location of each cluster in this climate category. The weekly median difference between the reference category climate traces and the actual climate traces at any given cluster was less than 3°C for temperature and within day-to-day stochastic variation in temperature, less than 0.11 for relative humidity and within day-to-day stochastic variation in relative humidity, and less than 12.33mm for rainfall and within day-to-day stochastic variation in rainfall, ensuring the climate input for the calibration of each node’s habitats matched the climate input at each node in spatial simulations.

During the calibration process, where cluster-level prevalence and seasonality of clinical case counts were fitted to data, each cluster simulation modeled an isolated population of 1000 people over a period of 8 years beginning in 2006, and importation due to migration between clusters was approximated by recurrent outbreaks every 180 days infecting 1% of the population.

#### Population immunity

After the establishment of the Lake Kariba reservoir in the 1960s, mosquito habitats and malaria prevalence grew. Acquired immunity over the antecedent 50 years will affect malaria transmission levels and clinical incidence during the study period. To characterize the age- and exposure-specific immunity of pre-intervention, pre-2006 population, each pair in the Cartesian product of larval habitat parameters at each of the four reference climates was simulated for 50 years in the absence of interventions.

#### Fitting prevalence and incidence in village-scale clusters

Both cluster-level prevalence by RDT from the six MTAT rounds in 2012–13 and the December 2011 pilot round as well as weekly clinical case counts from the Zambia National Malaria Control Centre’s Malaria Rapid Report system were used to inform the selection of *arabiensis* and *funestus* larval habitat availabilities at each cluster.

Across the region, fewer individuals aged 15 to 30 were surveyed than would be expected through equal representation of the standard Zambian demographics curve [41]. Cluster-specific population distributions, coupled with age-dependent prevalence, were found to bias the local mean RDT positivity compared to the simulations using fixed age tables (S7 Fig). A prevalence correction to adjust for this bias was made prior to comparing simulated and observed prevalence.

The simulated prevalence and clinical incidence for each pair of *arabiensis* and *funestus* larval habitat availabilities were fit to the observed data. Weekly clinical incidence data at HFCA level was obtained from the DHIS rapid-reporting system as RDT-positive fevers [42]. Fit quality was evaluated based on the weighted sum of mean square error of prevalence and rank correlation of clinical incidence.

Let *M*_*c*_ be the set of MTAT rounds administered in cluster *c*. The optimal fit pair, $s_{c}^{*}$, of *arabiensis* and *funestus* larval habitat availabilities for cluster *c* is

$$s_{c}^{*}=\underset{s\in S}{\mathrm{argmin}}\left(\alpha \sum_{i\in M_{c}}{w_{i}(\hat{{RDT}_{i}^{c}(s)}-{RDT}_{i}^{c})}^{2}+(1-\alpha )[1-\rho_{c}(\hat{{CC}^{c}(s)},{CC}^{c})]\right)$$ (2)

where *S* is the sampled space of *arabiensis* and *funestus* larval habitat availabilities; α is the relative weight of the two fit function components (prevalence and clinical incidence); *w*_*i*_ is the relative weight ascribed to each round of MTAT surveillance; $\hat{{RDT}_{i}^{c}(s)}$ and ${RDT}_{i}^{c}$ are respectively the modeled and observed RDT prevalence in cluster *c* at round *i* for habitat pair *s*; and $\rho_{c}(\hat{{CC}^{c}(s)},{CC}^{c})$ is the Spearman’s rank correlation coefficient [43] between the modeled $\hat{{CC}^{c}(s)}$ and observed *CC*^*c*^ clinical case timeseries at cluster *c*, with $-1\leq \rho_{c}(\hat{{CC}^{c}(s)},{CC}^{c})\leq 1$.

The round weight *w*_*i*_ describes the relative importance of each round for the fit. For example, since the pilot round is the only one in December, it provides important information about both magnitude and seasonality of malaria transmission. Relative round weights [3, 6, 1, 1, 6, 1, 1] were chosen respectively for the pilot round, the 1^st^, 2^nd^ and 3^rd^ rounds of the 2012–13 MTAT, and *w*_i_’s were calculated from relative round weights: $w_{i}=\frac{r_{i}}{\sum_{i\in M_{c}}r_{i}}$, where *i* ∈ *M*_*c*_. Data from the first round of each MTAT provides important information of malaria transmission magnitude immediately following a wet-season containing no interventions; these two rounds were respectively weighted more heavily.

To extract the yearly seasonality of weekly reported clinical cases at each cluster *c*, the observed and modeled timeseries were each aggregated in 6-week bins, and clinical case counts for each 6-week bin were aggregated and averaged over three years. The resulting smoothed clinical case timeseries were robust to missing weekly clinical-cases reports during the three-year interval. Since the observed weekly clinical cases were reported at the HFCA level, cluster-level clinical cases were scaled to account for 1) the probability each clinical case in a cluster would be treated and 2) the difference between the HFCA and cluster-level populations. The rank correlation provides a measure of curve-shapes’ similarity and captures the goodness of fit to the seasonality of clinical incidence. Note that the rank-correlation fit function term penalizes incongruities between observed and simulated trends in the seasonality of clinical cases but does not penalize differences in magnitude.

Setting α to higher values increases the importance of fit to prevalence at the expense of seasonality of clinical incidence. We prioritized minimizing error with respect to prevalence because 1) the seasonality of reported clinical cases may not be robust due to data collection errors, especially in some HFCAs; 2) clinical cases are reported at the HFCA level, so higher-resolution inter-cluster variation is smoothed; 3) our model of health seeking behavior does not necessarily reflect health-seeking patterns across all areas in the Lake Kariba region. We reduced alpha from 1 to 0.925 at increments of 0.0015 and picked an alpha that globally preserved the already good fits at the majority of clusters based on prevalence, but excised prevalence overfitting to subsets of rounds in most of the remaining clusters by taking into account the seasonality of clinical case counts. The model fit was satisfactory across all clusters for α = 0.9985. This global admixture of seasonality of clinical cases also adds overall protection to overfitting to the 3–7 prevalence data points at each cluster, as seen in results of sampling around the combined best fit to both prevalence and seasonality of clinical case counts (Fig 3 and S8 Fig).

### Spatial simulation of the Lake Kariba region

Given best-fit habitat parameters for each cluster along with cluster-specific climate, population, historical ITN usage, and drug campaign coverage, the set of 115 clusters was simulated together in a spatial model. Cluster climate was simulated using climate data at cluster’s centroid coordinates, and cluster population was set to the median population size of aggregated households within the cluster across the 2012–13 MTAT rounds. Total population over all 115 clusters was around 52,600 individuals, corresponding to the average number of individuals surveilled in each round rather than the total number of distinct individuals surveilled over all rounds. To account for population scaling, each cluster’s calibrated larval habitat parameters were proportionally adjusted to scale the magnitude of vector populations. A cluster of population *p* in the spatial simulation had larval habitat parameters adjusted to $\frac{p}{1000}s_{c}^{*}$. Importations of malaria from outside the study area were not included unless specifically indicated.

Simulations modeled a period of 24 years beginning in 2006, and climate data after 2013 was inferred from the preceding years. The baseline simulation up to and including the 2012–13 MTAT activities was extended to include the 2014–15 MDA interventions in the Lake Kariba region. Simulated MDA campaigns with dihydroartemisinin-piperaquine (DP) were administered to all clusters in two campaigns beginning in December 2014 and July 2015. Each MDA campaign consisted of two rounds separated by 60 days. During MDA, individuals received treatment regardless of their RDT result. The coverage of the MDA campaigns in each cluster was set to the coverage level of the 2012–13 MTAT campaign for that cluster. DP was administered with age-dependent dosing and full compliance with all treatment courses was assumed unless specifically indicated.

### Predicting outcomes of future interventions

Potential post-2015 intervention mixes were simulated to evaluate their ability to reach and maintain malaria elimination in the Lake Kariba region by 2030 (Figs 6–10), in line with southern Africa regional elimination goals. Combinations of ramp-ups in case management, increases in ITN usage, extending drug campaigns for an additional five years, targeting MDAs at high-burden HFCAs, coverage achieved by drug campaigns, impact of human migration rates, and impact of importation into the region were simulated and compared.

Case management ramp-up (scenarios 2–13) was modeled as gradual increase of both the percentage of people with access to malaria treatment and the rate at which symptomatic people seek treatment given treatment is available. Ramp-ups in case management were modeled to begin in January 2012 and plateau in January 2019 (S4C Fig), by which point 93% of malaria clinical cases in children under 5 received treatment, 89% of malaria clinical cases in people over age 5 received treatment and 98% of severe malaria cases across the entire population received treatment.

Three ITN coverage ramp-up options over the period 2014–22 were considered (S3B Fig and S11 Fig). Under “maintain current” (scenarios 1, 2, and 5), ITN coverage is maintained at 2015 levels via new ITNs distributed to individuals at birth; under “ramp-up” (scenarios 3, 6, 8, 10), ITN coverage is gradually increased between 2016 and 2022, when all distributions cease, extrapolating the historical and present ITN distribution coverage trajectory for each cluster post-2013; and under “aggressive” (scenarios 4, 7, 9, 11–13), ITN distributions covering 80% of the population administered every other year between 2016 and 2022.

For simulations where MDAs were extended through 2020 (scenarios 5–10, 12–13), five annual MDA campaigns with DP were administered beginning each July for the five years from 2016–20. Each campaign consisted of two rounds separated by 60 days. Campaign coverage at each cluster was set at the cluster’s 2012–13 MTAT coverage and did not vary from year to year.

In addition to MDAs over the entire Lake Kariba region, targeted MDA campaigns were simulated (scenarios 8, 9, 11). The twelve HFCAs were divided into high- and low-burden groups where high-burden HFCAs were those with RDT prevalence above 10% in children during surveillance in April 2014. In simulations of targeted MDAs, clusters in high-burden HFCAs received five additional annual MDAs with DP beginning in July 2016 as described above, while clusters in low-burden HFCAs did not receive MDA after the 2014–15 rounds.

All the intervention mix scenarios were simulated in the context of human migration across clusters. Scenarios were simulated at two migration settings. For scenarios 1–11 and 13, low migration, 7,440 round trips and 220 permanent relocations occurred each year. For scenario 12, high migration, 74,400 round trips and 2,200 permanent relocations occurred each year. In all scenarios, migration was spread evenly throughout the year and was independent of age.

Other than scenario 13, no intervention scenarios included importation of infections from outside the study area. For scenario 13, a total of 100 infections were imported annually into the entire study area. Three highly connected, high-population clusters, one from each of Gwembe, Munyumbwe, and Sinamalima HFCAs, were selected to be the loci of importation.

## Supporting Information

S1 Dataset Surveyed cluster prevalence by round. (CSV)

S1 Table Selection criteria for longitudinally linking individuals. (DOCX)

S1 Fig Surveillance and simulation demographics. (A) Simulation demographics approximate the age distribution observed in surveillance data. Compared with simulation, surveillance data is missing a portion of 15–30 year old adults who may not have been at home during the MSAT rounds. (B) Distribution of cluster populations used in spatial simulation framework. See Fig 1 for cluster locations.
(PDF)

S2 Fig Spatial and seasonal variation in climate used in simulation. (A) Temperature varies spatially within the study region. Shown: June 1, 2005 and December 1, 2005 daily average temperature. (B) Annual variation in daily temperature, rainfall, and humidity shows a warm, wet season in October through April and a cool, dry season in June through October. Shown: 2005 climate series for a representative cluster in Munyumbwe HFCA. (C) During calibration, clusters are assigned to a reference climate category based on cluster altitude.
(PDF)

S3 Fig ITN usage in surveillance and simulation. (A) Cluster-level ITN usage estimates from fraction of cluster population who used a net last night in survey data. ITN usage in the study area varies both spatially and seasonally. (B) Clusters are assigned one of four ITN usage levels in simulation, and each level is associated with a ramp-up trajectory between 2005 and 2015 based on coverage estimates from the Malaria Atlas Project. (C) Age-dependence of ITN usage in survey data and simulation (spline fits). (D) ITN effects are similar in survey and simulation. Fold-change in risk is probability of testing RDT positive for people not using ITNs compared with those who do. Dots and triangles: individual round data from individual clusters. Line: spline fit.
(PDF)

S4 Fig Health-seeking in surveillance and simulation. (A) Proportion with fever in previous two weeks who sought health services for their fever varies with age. Spline fit to surveillance data, all clusters, all rounds. (B) Fraction of fevers that sought care across six rounds of surveillance depends on distance to the local health facility. These cluster-level case management rates were only used as scale factors when comparing simulated clinical cases with weekly reported RDT+ fevers in Fig 5. During simulation, case management rates were uniform across all clusters. (C) Case management rates for clinical and severe disease used in simulation. All clusters were simulated with the same case management rates. Hypothetical ramp-ups in case management used in intervention scenarios in Fig 4 begin in 2015 and plateau in 2022.
(PDF)

S5 Fig MTAT coverage by HFCA. (A) Coverage estimated from linked individuals in surveillance data. (B) MTAT and MDA coverage used in simulation.
(PDF)

S6 Fig MTAT coverage can vary substantially within an HFCA. As an example, the Luumbo HFCA was gridded and the number of individuals in each grid square at each round was counted. Totals were normalized by the maximum count for a square across rounds. Although there is some patchiness in coverage, in that some places are very well surveyed and others are not visited during some rounds at all, there are also areas where only a fraction of the population was censed in a given round.
(PDF)

S7 Fig Prevalence correction based on differences in demographics between surveillance data and simulation. In examining the demographics distributions from the study, many clusters appeared to have reduced participation among adults compared to children. Thus, simulated prevalence values may underestimate those seen in the study, which are biased to younger populations with higher prevalence compared to adults in the same cluster. New prevalence estimates were computed for each cluster under the assumption that the age distribution followed the modeled demographics rather than the actual ones. The difference in study vs. model prevalence increases steadily with endemicity, with a bias of about 0.05 at a modeled prevalence of 0.4. In the above scatterplot, clusters are colored by their HFCA. Demographics curves and corresponding difference in prevalence are shown for a representative cluster in Sinamalima HFCA.
(PDF)

S8 Fig Example calibration of a cluster where best fits to prevalence and clinical cases show somewhat different patterns of seasonality. See caption for Fig 3 for details.
(PDF)

S9 Fig Surveillance data and simulated cluster RDT prevalence across six MTAT rounds. (PDF)

S10 Fig Out-of-sample prediction of December 2014 prevalence by RDT with the full spatial simulation. Surveillance data was available for 8 out of 12 HFCAs. Dot size in scatter plot indicates relative cluster population. Line is y = x. To mirror interventions conducted in the area, simulations included ramp-up in case management, ITN mass distribution in July 2014, and a rudimentary reactive case detection where a random 10 individuals in a cluster are given AL when someone in the cluster receives treatment for clinical malaria.
(PDF)

S11 Fig ITN ramp-up trajectories used in post-2015 intervention scenarios. (A) ITN usage rates by age in 2015 and 2020 after ramp-up and aggressive distributions shown in S11C Fig and S11E Fig. (B) Usage rates of ITNs newer than 3 years old under the “maintain current coverage” scenario shown in S3B Fig. The spikes are due to the 1080-day interim between net distributions. (C, D) Usage rates of ITNs under the “ramp-up” scenario: (C) usage of any net and (D) usage of nets newer than 3 years old. (E, F) Usage rates of ITNs under the “aggressive” scenario: (E) usage of any net and (F) usage of nets newer than 3 years old.
(PDF)

S12 Fig When elimination fails, transmission is re-established in high-population, high-transmission clusters. Single simulation of ramp-up in case management, aggressive ITN distribution, and 5 years of post-2015 MDA at historical coverage levels (S6 Fig) in all HFCAs.
(PDF)

S13 Fig Impact of importations with and without routine vector control interventions. (A) Representative single simulation of scenario 13, where repeated importation of infections into the region results in reestablishment of transmission after vector control and MDAs cease in 2022. Prior to 2030, nearly all transmission is still localized to Sinamalima HFCA even though importations are also occurring in Munyumbwe and Gwembe HFCAs. (B) Maintaining vector control in areas with imported infections prevents reestablishment of transmission. In the yellow scenario, annual IRS campaigns with long-lasting insecticide (initial efficacy 0.6, half-life 9 months) at 75% coverage are distributed in all Sinamalima and Munyumbwe clusters between December 2022 and December 2029.
(PDF)

S14 Fig Elimination is equally likely under extremely poor compliance with MDA treatment when ITN coverage is aggressively increased. In these simulations, all individuals receiving DP as part of an MDA take only the first dose of the three-dose treatment regimen.
(PDF)

## References

[1] World Health Organization. World Malaria Report 2015 Geneva: World Health Organization, 2015.

[2] Bhatt S, Weiss DJ, Cameron E, et al The effect of malaria control on *Plasmodium falciparum* in Africa between 2000 and 2015. Nature 2015; 526: 207–11. 10.1038/nature15535 26375008 PMC4820050

[3] Poirot E, Skarbinski J, Sinclair D, Kachur SP, Slutsker L, Hwang J. Mass drug administration for malaria. Cochrane Database Syst Rev 2013; 12: CD008846 10.1002/14651858.CD008846.pub2 24318836 PMC4468927

[4] Tatem AJ, Smith DL, Gething PW, Kabaria CW, Snow RW, Hay SI. Ranking of elimination feasibility between malaria-endemic countries. Lancet 2010; 376: 1579–91. 10.1016/S0140-6736(10)61301-3 21035838 PMC3044847

[5] Tatem AJ, Smith DL. International population movements and regional *Plasmodium falciparum* malaria elimination strategies. Proc Natl Acad Sci U S A 2010; 107: 12222–7. 10.1073/pnas.1002971107 20566870 PMC2901446

[6] Cotter C, Sturrock HJ, Hsiang MS, et al The changing epidemiology of malaria elimination: new strategies for new challenges. Lancet 2013; 382: 900–11. 10.1016/S0140-6736(13)60310-4 23594387 PMC10583787

[7] Larsen DA, Bennett A, Silumbe K, et al Population-Wide Malaria Testing and Treatment with Rapid Diagnostic Tests and Artemether-Lumefantrine in Southern Zambia: A Community Randomized Step-Wedge Control Trial Design. Am J Trop Med Hyg 2015; 92: 913–21. 10.4269/ajtmh.14-0347 25802434 PMC4426577

[8] Larsen DA, Chisha Z, Winters B, et al Malaria surveillance in low-transmission areas of Zambia using reactive case detection. Malar J 2015; 14: 465 10.1186/s12936-015-0895-9 26586264 PMC4653936

[9] Gerardin J, Bever CA, Hamainza B, Miller JM, Eckhoff PA, Wenger EA. Optimal Population-Level Infection Detection Strategies for Malaria Control and Elimination in a Spatial Model of Malaria Transmission. PLoS Comput Biol 2016; 12(1): e1004707 10.1371/journal.pcbi.1004707 26764905 PMC4713231

[10] Griffin JT, Hollingsworth TD, Okell LC, et al Reducing *Plasmodium falciparum* Malaria Transmission in Africa: A Model-Based Evaluation of Intervention Strategies. PLoS Med 2010; 7: e1000324 10.1371/journal.pmed.1000324 20711482 PMC2919425

[11] Gerardin J, Eckhoff P, Wenger EA. Mass campaigns with antimalarial drugs: a modelling comparison of artemether-lumefantrine and DHA-piperaquine with and without primaquine as tools for malaria control and elimination. BMC Infect Dis 2015; 15: 144 10.1186/s12879-015-0887-y 25887935 PMC4376519

[12] Maude RJ, Socheat D, Nguon C, et al Optimising strategies for *Plasmodium falciparum* malaria elimination in Cambodia: primaquine, mass drug administration and artemisinin resistance. PLoS One 2012; 7: e37166 10.1371/journal.pone.0037166 22662135 PMC3360685

[13] Gu W, Killeen GF, Mbogo CM, Regens JL, Githure JI, Beier JC. An individual-based model of *Plasmodium falciparum* malaria transmission on the coast of Kenya. Trans R Soc Trop Med Hyg 2003; 97: 43–50. 12886804 10.1016/s0035-9203(03)90018-6

[14] Silal SP, Little F, Barnes KI, White LJ. Hitting a Moving Target: A Model for Malaria Elimination in the Presence of Population Movement. PLoS One 2015; 10: e0144990 10.1371/journal.pone.0144990 26689547 PMC4686217

[15] Killeen GF, McKenzie FE, Foy BD, Bøgh C, Beier JC. The availability of potential hosts as a determinant of feeding behaviours and malaria transmission by African mosquito populations. Trans R Soc Trop Med Hyg 2001; 95: 469–76. 11706651 10.1016/s0035-9203(01)90005-7 PMC2483839

[16] Okell LC, Bousema T, Griffin JT, Ouédraogo AL, Ghani AC, Drakeley CJ. Factors determining the occurrence of submicroscopic malaria infections and their relevance for control. Nature Comm 2012; 3: 1237. 10.1038/ncomms2241 PMC3535331 23212366

[17] Tiono AB, Ouédraogo A, Ogutu B, et al A controlled, parallel, cluster-randomized trial of community-wide screening and treatment of asymptomatic carriers of *Plasmodium falciparum* in Burkina Faso. Malar J 2013; 12: 79 10.1186/1475-2875-12-79 23442748 PMC3599538

[18] Gerardin J, Ouédraogo AL, McCarthy KA, Eckhoff PA, Wenger EA. Characterization of the infectious reservoir of malaria with an agent-based model calibrated to age-stratified parasite densities and infectiousness. Malar J 2015; 14: 231 10.1186/s12936-015-0751-y 26037226 PMC4702301

[19] Slater HC, Ross A, Ouédraogo AL, et al Assessing the impact of next-generation rapid diagnostic tests on *Plasmodium falciparum* malaria elimination strategies. Nature 2015; 528: S94–S101. 10.1038/nature16040 26633771

[20] Halliday KE, Okello G, Turner EL, et al Impact of Intermittent Screening and Treatment for Malaria among School Children in Kenya: A Cluster Randomised Trial. PLoS Med 2014; 11: e1001594 10.1371/journal.pmed.1001594 24492859 PMC3904819

[21] Kern SE, Tiono AB, Makanga M, et al Community screening and treatment of a symptomatic carriers of *Plasmodium falciparum* with artemether-lumefantrine to reduce malaria disease burden: a modelling and simulation analysis. Malar J 2011; 10: 210 10.1186/1475-2875-10-210 21801345 PMC3161019

[22] Marshall JM, Mahamoudou BT, Ouédraogo AL, Ndhlovu M, Kiware SS, Rezai A, et al Key traveler groups of relevance to spatial malaria transmission: a survey of movement patterns in four sub-Saharan African countries. Malar J 2016; 15: 200 10.1186/s12936-016-1252-3 27068686 PMC4828820

[23] Wesolowski A, Eagle N, Tatem AJ, Smith DL, Noor AM, Snow RW, et al Quantifying the impact of human mobility on malaria. Science 2012; 338: 267 10.1126/science.1223467 23066082 PMC3675794

[24] Ruktanonchai NW, DeLeenheer P, Tatem AJ, Alegana VA, Caughlin TT, zu Erbach-Shoenberg E, et al Identifying malaria transmission foci for elimination using human mobility data. PLoS Comput Biol 2016; 12(4): e1004846 10.1371/journal.pcbi.1004846 27043913 PMC4820264

[25] Yeung S, White NJ. How do patients use antimalarial drugs? A review of the evidence. Trop Med Int Health 2005; 10: 121–38. 10.1111/j.1365-3156.2004.01364.x 15679555

[26] Minzi O, Maige S, Sasi P, Ngasala B. Adherence to artemether-lumefantrine drug combination: a rural community experience six years after change of malaria treatment policy in Tanzania. Malar J 2014; 13: 267 10.1186/1475-2875-13-267 25011682 PMC4105528

[27] Briët OJ, Penny MA. Repeated mass distributions and continuous distribution of long-lasting insecticidal nets: modelling sustainability of health benefits from mosquito nets, depending on case management. Malar J 2013; 12: 401 10.1186/1475-2875-12-401 24200296 PMC4228503

[28] Bousema T, Griffin JT, Sauerwein RW, et al Hitting Hotspots: Spatial Targeting of Malaria for Control and Elimination. PLoS Med 2012; 9: e1001165 10.1371/journal.pmed.1001165 22303287 PMC3269430

[29] Eckhoff PA. A malaria transmission-directed model of mosquito life cycle and ecology. Malar J 2011; 10: 303 10.1186/1475-2875-10-303 21999664 PMC3224385

[30] Eckhoff P. *P*. *falciparum* Infection Durations and Infectiousness Are Shaped by Antigenic Variation and Innate and Adaptive Host Immunity in a Mathematical Model. PLoS One 2012; 7: e44950 10.1371/journal.pone.0044950 23028698 PMC3446976

[31] Eckhoff PA. Malaria parasite diversity and transmission intensity affect development of parasitological immunity in a mathematical model. Malar J 2012; 11: 419 10.1186/1475-2875-11-419 23241282 PMC3557182

[32] Eckhoff P. Mathematical Models of Within-Host and Transmission Dynamics to Determine Effects of Malaria Interventions in a Variety of Transmission Settings. Am J Trop Med Hyg 2013; 88: 817–27. 10.4269/ajtmh.12-0007 23589530 PMC3752743

[33] Institute for Disease Modeling. Epidemiological Modeling Software. 2015.

[34] McCarthy KA, Wenger EA, Huynh GH, Eckhoff PA. Calibration of an intrahost malaria model and parameter ensemble evaluation of a pre-erythrocytic vaccine. Malar J 2015; 14: 6 10.1186/1475-2875-14-6 25563798 PMC4326442

[35] Chabot-Couture G, Nigmatulina K, Eckhoff P. An Environmental Data Set for Vector-Borne Disease Modeling and Epidemiology. PLoS ONE 2014; 9: e94741 10.1371/journal.pone.0094741 24755954 PMC3995884

[36] Rainfall Estimator 2.0. http://www.cpc.ncep.noaa.gov/products/fews/data.shtml (accessed July 15, 2015).

[37] Bhatt S, Weiss DJ, Mappin B, et al Coverage and system efficiencies of insecticide-treated nets in Africa from 2000 to 2017. eLife 2015; 4. 10.7554/eLife.09672 PMC4758960 26714109

[38] Seyoum A, Sikaala CH, Chanda J, et al Human exposure to anopheline mosquitoes occurs primarily indoors, even for users of insecticide-treated nets in Luangwa Valley, South-east Zambia. Parasites Vectors 2012; 5: 101 10.1186/1756-3305-5-101 22647493 PMC3432592

[39] Fornadel CM, Norris LC, Glass GE, Norris DE. Analysis of Anopheles arabiensis Blood Feeding Behavior in Southern Zambia during the Two Years after Introduction of Insecticide-Treated Bed Nets. Am J Trop Med Hyg 2010; 83: 848–53. 10.4269/ajtmh.2010.10-0242 20889878 PMC2946755

[40] Norris LC, Norris DE. Efficacy of long-lasting insecticidal nets in use in Macha, Zambia, against the local *Anopheles arabiensis* population. Malar J 2011; 10: 254 10.1186/1475-2875-10-254 21880143 PMC3175477

[41] Global Health Observatory data repository. World Health Organization http://apps.who.int/gho/data/view.main.61850 (accessed November 12, 2015).

[42] Zambia National Malaria Control Centre DHIS. https://www.dhis.co.zm/dhis (accessed January 11, 2016).

[43] Zwillinger D, Kokoska S. CRC Standard Probability and Statistics Tables and Formulae Chapman & Hall: New York, 2000 Section 14.7: 369–70.

