# Supplementary material for: Malaria Elimination Campaigns in the Lake Kariba Region of Zambia: A Spatial Dynamical Model
Source: PLoS Comput Biol. 2016 Nov 23;12(11):e1005192. doi: 10.1371/journal.pcbi.1005192 (PMC5120780; doi:10.1371/journal.pcbi.1005192)
Supplement: S1 Table — (DOCX) [file pcbi.1005192.s002.docx]

**S1 Table. Selection criteria for longitudinally linking individuals**

|  | **Standard threshold** | **Restrictive threshold** |
| --- | --- | --- |
| Age difference | 2 years | 1 year |
| Levenshtein distance, first name | 3 | 1 |
| Levenshtein distance, last name | 3 | 1 |
| Geographical distance | 250m | 150m |
