# Supplementary material for: Malaria Elimination Campaigns in the Lake Kariba Region of Zambia: A Spatial Dynamical Model
Source: PLoS Comput Biol. 2016 Nov 23;12(11):e1005192. doi: 10.1371/journal.pcbi.1005192 (PMC5120780; doi:10.1371/journal.pcbi.1005192)
Supplement: S1 Fig — (A) Simulation demographics approximate the age distribution observed in surveillance data. Compared with simulation, surveillance data is missing a portion of 15–30 year old adults who may not have been at home during the MSAT rounds. (B) Distribution of cluster populations used in spatial simulation framework. See Fig 1 for cluster locations. (PDF) [file pcbi.1005192.s003.pdf]

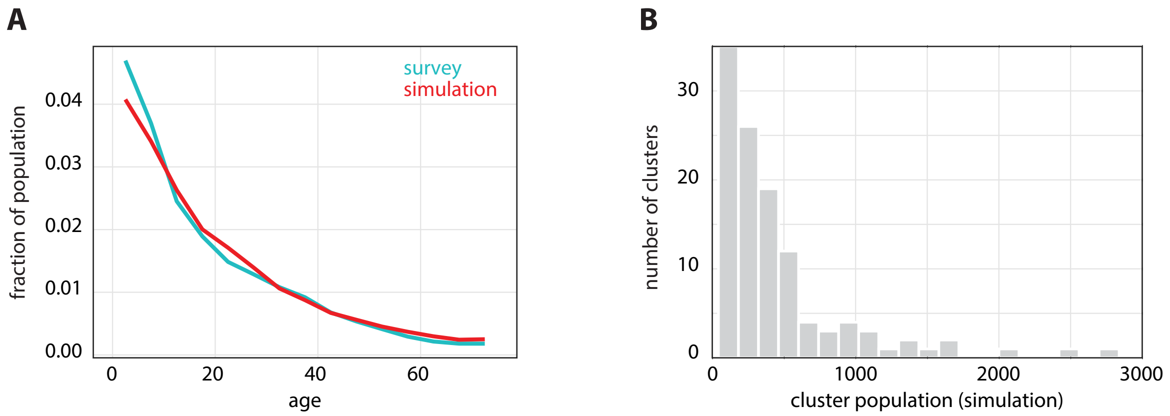


Figure S1. Surveillance and simulation demographics. (A) Simulation demographics approximate the age distribution observed in surveillance data. Compared with simulation, surveillance data is missing a portion of 15-30 year old adults who may not have been at home during the MSAT rounds. (B) Distribution of cluster populations used in spatial simulation framework. See Figure 1 for cluster locations.
