# Supplementary material for: Malaria Elimination Campaigns in the Lake Kariba Region of Zambia: A Spatial Dynamical Model
Source: PLoS Comput Biol. 2016 Nov 23;12(11):e1005192. doi: 10.1371/journal.pcbi.1005192 (PMC5120780; doi:10.1371/journal.pcbi.1005192)
Supplement: S2 Fig — (A) Temperature varies spatially within the study region. Shown: June 1, 2005 and December 1, 2005 daily average temperature. (B) Annual variation in daily temperature, rainfall, and humidity shows a warm, wet season in October through April and a cool, dry season in June through October. Shown: 2005 climate series for a representative cluster in Munyumbwe HFCA. (C) During calibration, clusters are assigned to a reference climate category based on cluster altitude. (PDF) [file pcbi.1005192.s004.pdf]

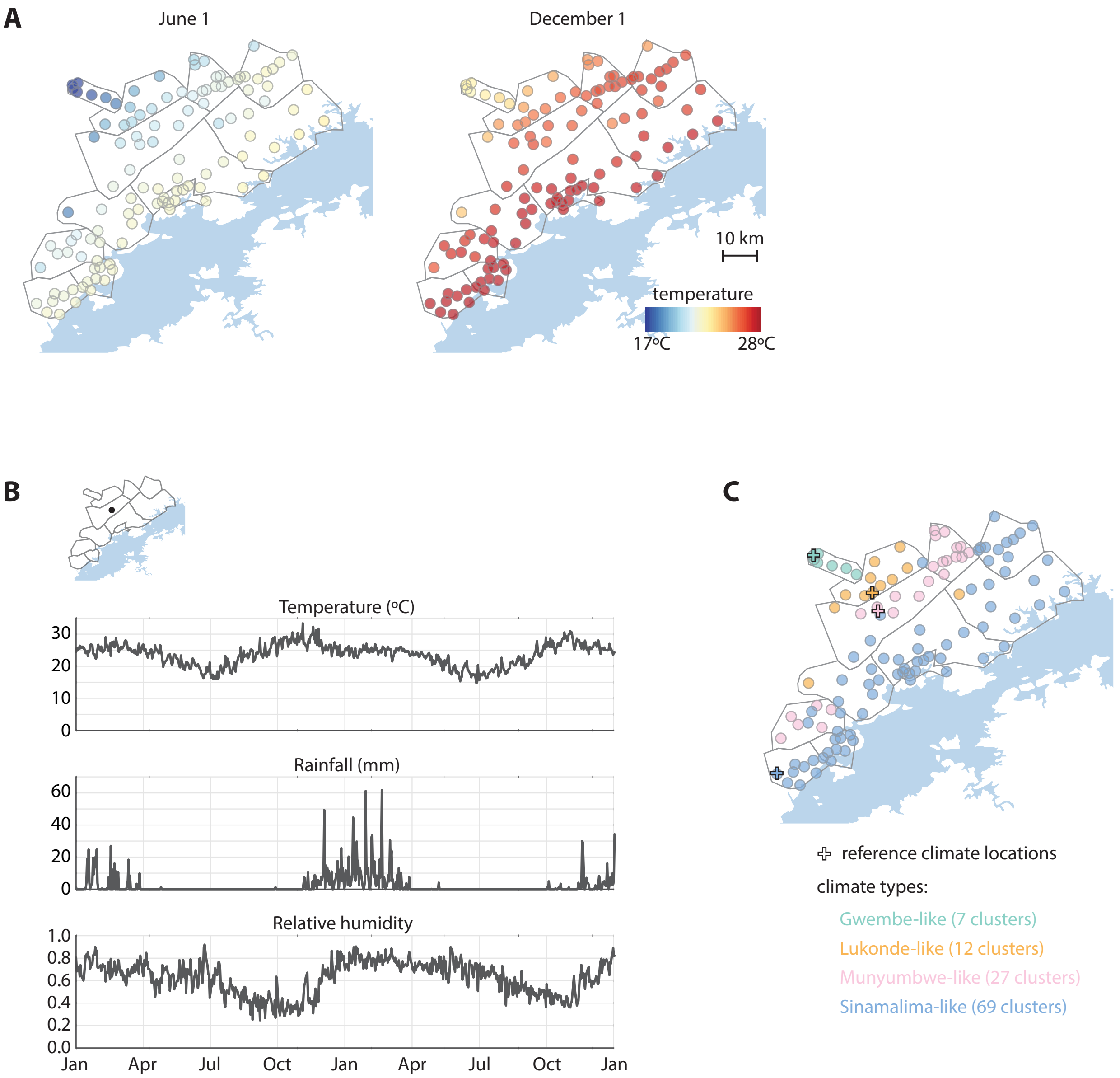

Figure S2. Spatial and seasonal variation in climate used in simulation. (A) Temperature varies spatially within the study region. Shown: June 1, 2005 and December 1, 2005 daily average temperature. (B) Annual variation in daily temperature, rainfall, and humidity shows a warm, wet season in October through April and a cool, dry season in June through October. Shown: 2005 climate series for a representative cluster in Munyumbwe HFCA. (C) During calibration, clusters are assigned to a reference climate category based on cluster altitude.
