# Supplementary material for: Malaria Elimination Campaigns in the Lake Kariba Region of Zambia: A Spatial Dynamical Model
Source: PLoS Comput Biol. 2016 Nov 23;12(11):e1005192. doi: 10.1371/journal.pcbi.1005192 (PMC5120780; doi:10.1371/journal.pcbi.1005192)
Supplement: S3 Fig — (A) Cluster-level ITN usage estimates from fraction of cluster population who used a net last night in survey data. ITN usage in the study area varies both spatially and seasonally. (B) Clusters are assigned one of four ITN usage levels in simulation, and each level is associated with a ramp-up trajectory between 2005 and 2015 based on coverage estimates from the Malaria Atlas Project. (C) Age-dependence of ITN usage in survey data and simulation (spline fits). (D) ITN effects are similar in survey and simulation. Fold-change in risk is probability of testing RDT positive for people not using ITNs compared with those who do. Dots and triangles: individual round data from individual clusters. Line: spline fit. (PDF) [file pcbi.1005192.s005.pdf]

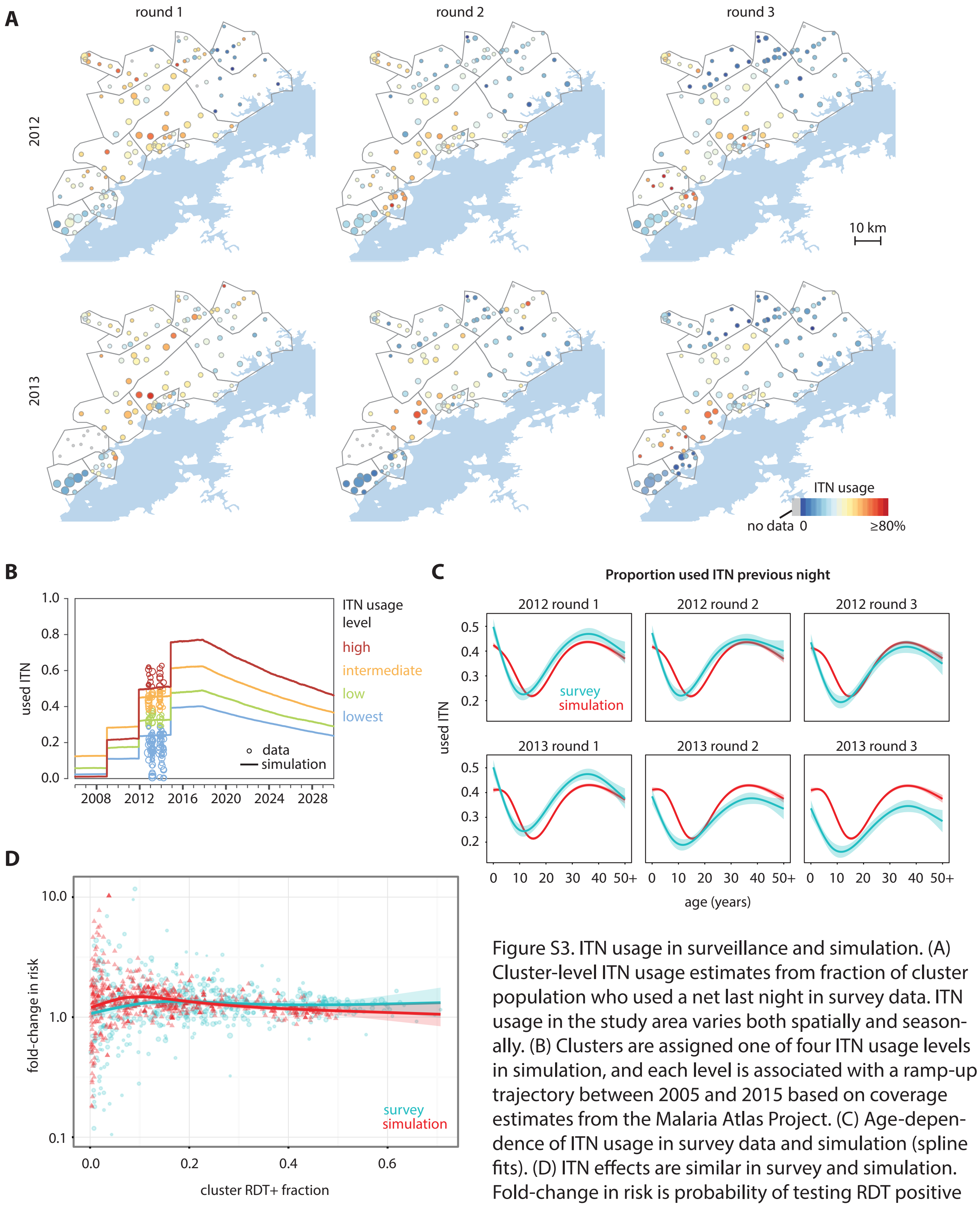

Figure S3. ITN usage in surveillance and simulation. (A) Cluster-level ITN usage estimates from fraction of cluster population who used a net last night in survey data. ITN usage in the study area varies both spatially and seasonally. (B) Clusters are assigned one of four ITN usage levels in simulation, and each level is associated with a ramp-up trajectory between 2005 and 2015 based on coverage estimates from the Malaria Atlas Project. (C) Age-dependence of ITN usage in survey data and simulation (spline fits). (D) ITN effects are similar in survey and simulation. Fold-change in risk is probability of testing RDT positive for people not using ITNs compared with those who do. Dots and triangles: individual round data from individual clusters. Line: spline fit.
