# Supplementary material for: Malaria Elimination Campaigns in the Lake Kariba Region of Zambia: A Spatial Dynamical Model
Source: PLoS Comput Biol. 2016 Nov 23;12(11):e1005192. doi: 10.1371/journal.pcbi.1005192 (PMC5120780; doi:10.1371/journal.pcbi.1005192)
Supplement: S4 Fig — (A) Proportion with fever in previous two weeks who sought health services for their fever varies with age. Spline fit to surveillance data, all clusters, all rounds. (B) Fraction of fevers that sought care across six rounds of surveillance depends on distance to the local health facility. These cluster-level case management rates were only used as scale factors when comparing simulated clinical cases with weekly reported RDT+ fevers in Fig 5. During simulation, case management rates were uniform across all clusters. (C) Case management rates for clinical and severe disease used in simulation. All clusters were simulated with the same case management rates. Hypothetical ramp-ups in case management used in intervention scenarios in Fig 4 begin in 2015 and plateau in 2022. (PDF) [file pcbi.1005192.s006.pdf]

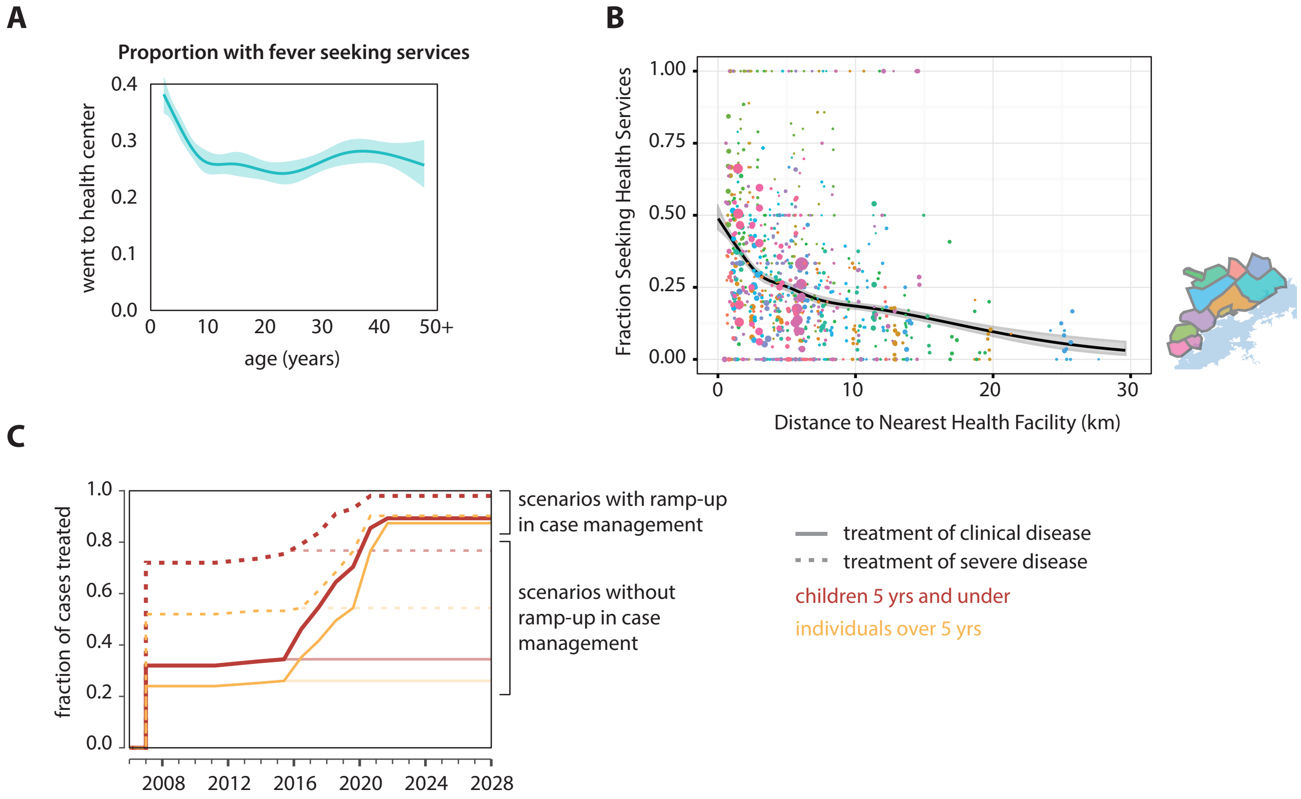

Figure S4. Health-seeking in surveillance and simulation.
(A) Proportion with fever in previous two weeks who sought health services for their fever varies with age. Spline fit to surveillance data, all clusters, all rounds.
(B) Fraction of fevers that sought care across six rounds of surveillance depends on distance to the local health facility. These cluster-level case management rates were only used as scale factors when comparing simulated clinical cases with weekly reported RDT+ fevers in Fig 5. During simulation, case management rates were uniform across all clusters.
(C) Case management rates for clinical and severe disease used in simulation. All clusters were simulated with the same case management rates. Hypothetical ramp-ups in case management used in intervention scenarios in Figure 4 begin in 2015 and plateau in 2022.
