# Supplementary material for: Malaria Elimination Campaigns in the Lake Kariba Region of Zambia: A Spatial Dynamical Model
Source: PLoS Comput Biol. 2016 Nov 23;12(11):e1005192. doi: 10.1371/journal.pcbi.1005192 (PMC5120780; doi:10.1371/journal.pcbi.1005192)
Supplement: S5 Fig — (A) Coverage estimated from linked individuals in surveillance data. (B) MTAT and MDA coverage used in simulation. (PDF) [file pcbi.1005192.s007.pdf]

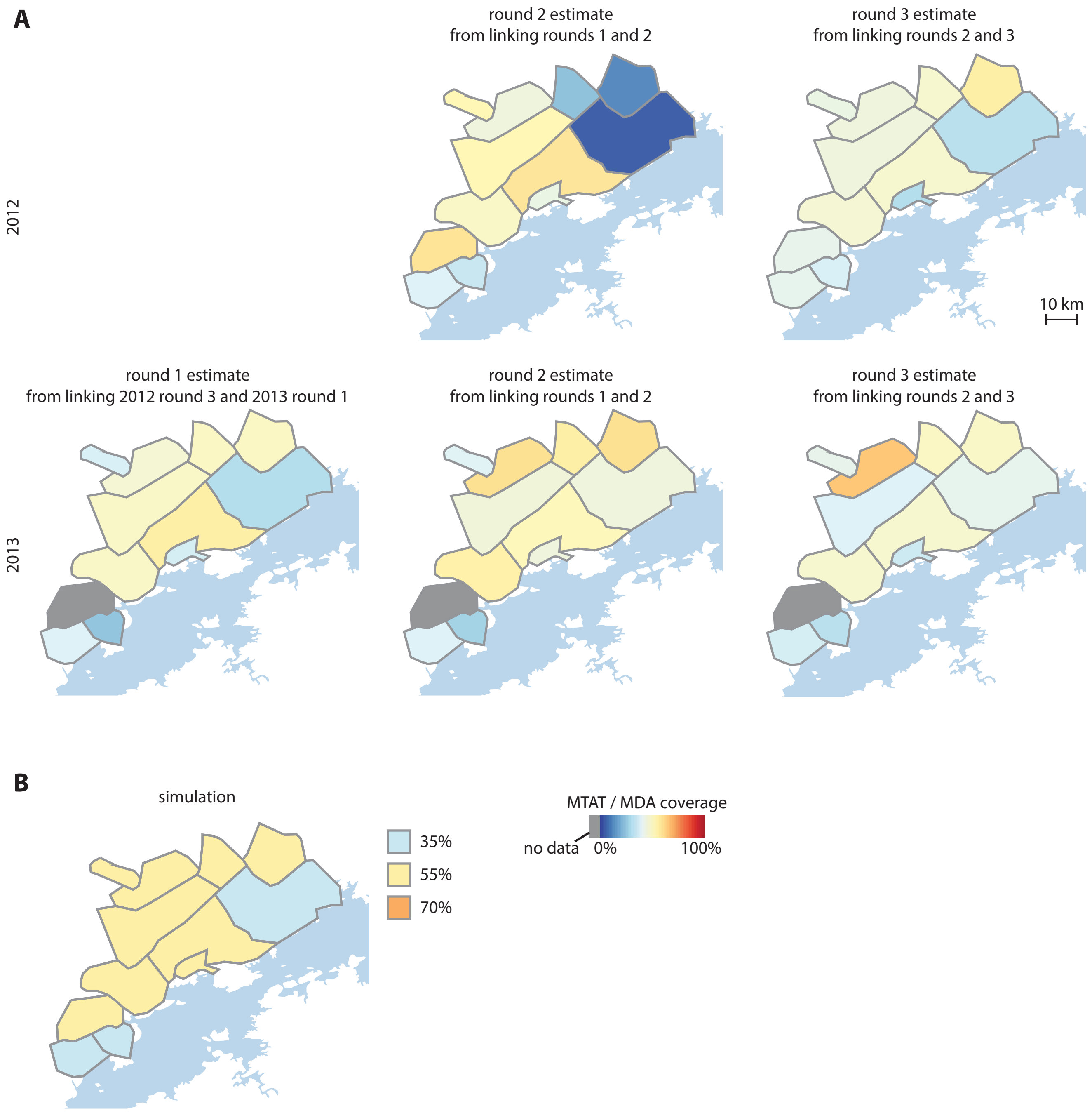

Figure S5. MTAT coverage by HFCA. (A) Coverage estimated from linked individuals in surveillance data. (B) MTAT and MDA coverage used in simulation.
