# Supplementary material for: Malaria Elimination Campaigns in the Lake Kariba Region of Zambia: A Spatial Dynamical Model
Source: PLoS Comput Biol. 2016 Nov 23;12(11):e1005192. doi: 10.1371/journal.pcbi.1005192 (PMC5120780; doi:10.1371/journal.pcbi.1005192)
Supplement: S6 Fig — As an example, the Luumbo HFCA was gridded and the number of individuals in each grid square at each round was counted. Totals were normalized by the maximum count for a square across rounds. Although there is some patchiness in coverage, in that some places are very well surveyed and others are not visited during some rounds at all, there are also areas where only a fraction of the population was censed in a given round. (PDF) [file pcbi.1005192.s008.pdf]

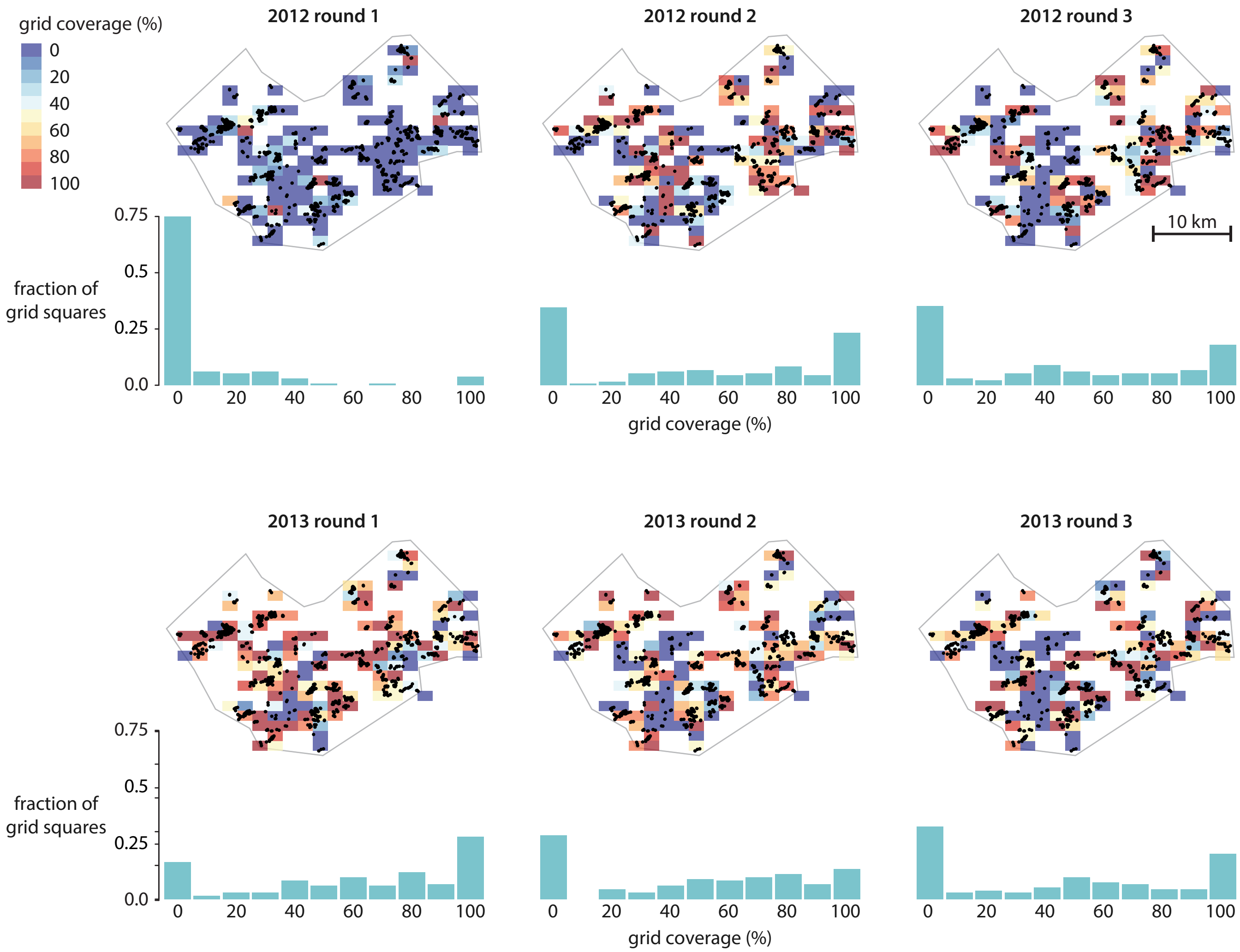

Figure S6. MSAT coverage can vary substantially within an HFCA. As an example, the Luumbo HFCA was gridded and the number of individuals in each grid square at each round was counted. Totals were normalized by the maximum count for a square across rounds. Although there is some patchiness in coverage, in that some places are very well surveyed and others are not visited during some rounds at all, there are also areas where only a fraction of the population was censed in a given round.
