# Supplementary material for: Malaria Elimination Campaigns in the Lake Kariba Region of Zambia: A Spatial Dynamical Model
Source: PLoS Comput Biol. 2016 Nov 23;12(11):e1005192. doi: 10.1371/journal.pcbi.1005192 (PMC5120780; doi:10.1371/journal.pcbi.1005192)
Supplement: S7 Fig — In examining the demographics distributions from the study, many clusters appeared to have reduced participation among adults compared to children. Thus, simulated prevalence values may underestimate those seen in the study, which are biased to younger populations with higher prevalence compared to adults in the same cluster. New prevalence estimates were computed for each cluster under the assumption that the age distribution followed the modeled demographics rather than the actual ones. The difference in study vs. model prevalence increases steadily with endemicity, with a bias of about 0.05 at a modeled prevalence of 0.4. In the above scatterplot, clusters are colored by their HFCA. Demographics curves and corresponding difference in prevalence are shown for a representative cluster in Sinamalima HFCA. (PDF) [file pcbi.1005192.s009.pdf]

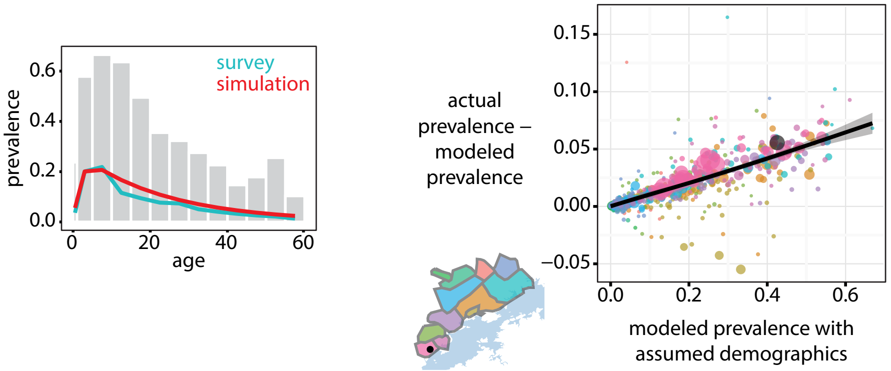

Figure S7. Prevalence correction based on differences in demographics between surveillance data and simulation. In examining the demographics distributions from the study, many clusters appeared to have reduced participation among adults compared to children. Thus, simulated prevalence values may underestimate those seen in the study, which are biased to younger populations with higher prevalence compared to adults in the same cluster. New prevalence estimates were computed for each cluster under the assumption that the age distribution followed the modeled demographics rather than the actual ones. The difference in study vs. model prevalence increases steadily with endemicity, with a bias of about 0.05 at a modeled prevalence of 0.4. In the above scatterplot, clusters are colored by their HFCA. Demographics curves and corresponding difference in prevalence are shown for a representative cluster in Sinamalima HFCA.
