# Supplementary material for: Malaria Elimination Campaigns in the Lake Kariba Region of Zambia: A Spatial Dynamical Model
Source: PLoS Comput Biol. 2016 Nov 23;12(11):e1005192. doi: 10.1371/journal.pcbi.1005192 (PMC5120780; doi:10.1371/journal.pcbi.1005192)
Supplement: S8 Fig — See caption for Fig 3 for details. (PDF) [file pcbi.1005192.s010.pdf]

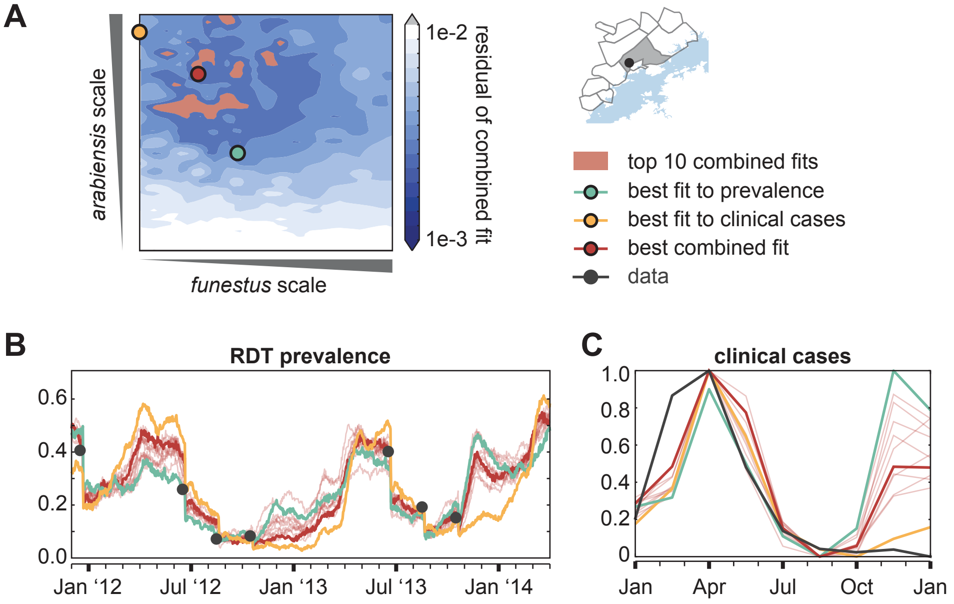

Figure S8. Example calibration of a cluster where best fits to prevalence and clinical cases show somwehat different patterns of seasonality. See caption for Fig 3 for details.
