# Supplementary figures and images for: Malaria Elimination Campaigns in the Lake Kariba Region of Zambia: A Spatial Dynamical Model
Source: PLoS Comput Biol. 2016 Nov 23;12(11):e1005192. doi: 10.1371/journal.pcbi.1005192 (PMC5120780; doi:10.1371/journal.pcbi.1005192)

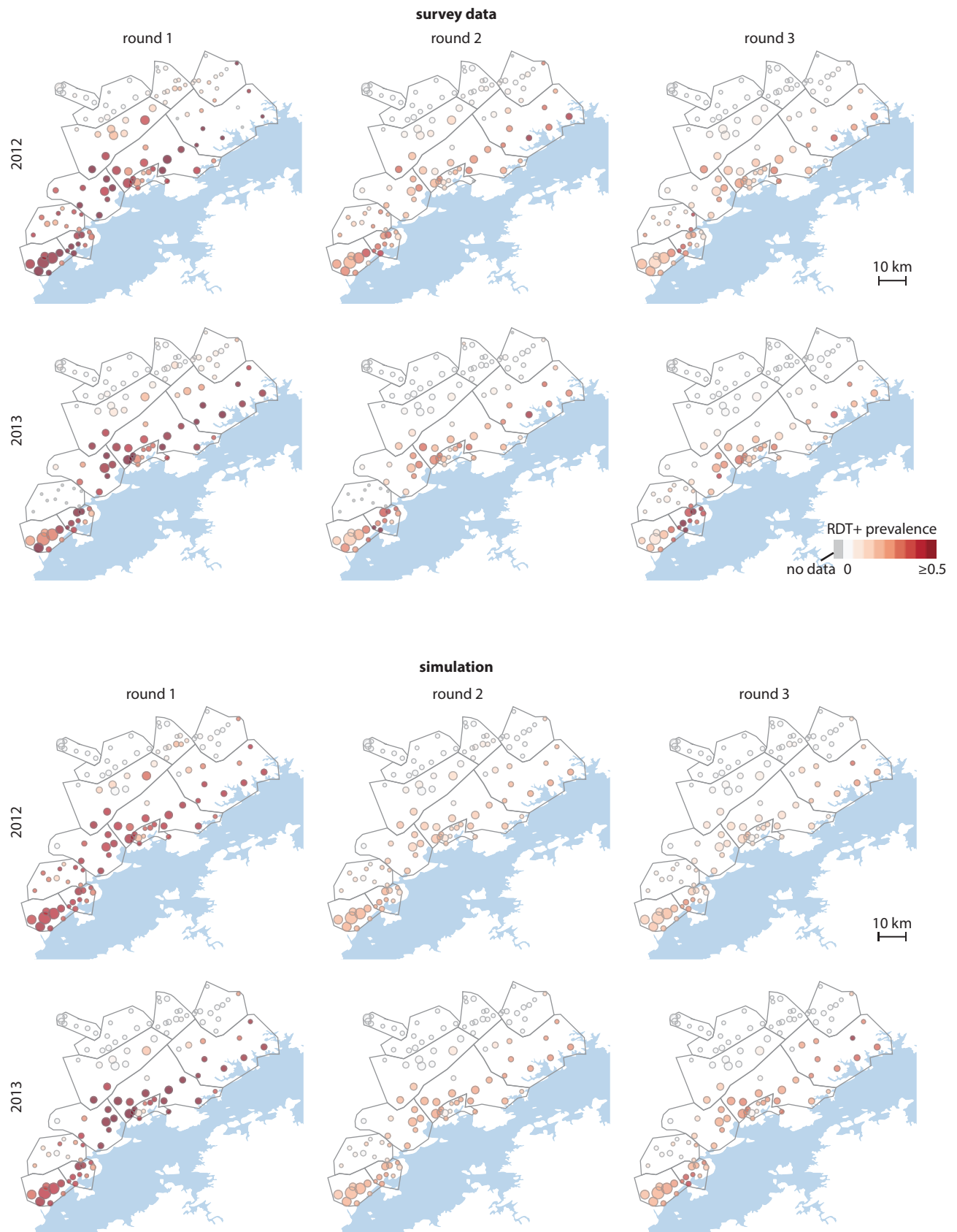

Figure S9. Surveillance data and simulated cluster RDT prevalence across six MSAT rounds.

Supplement: S9 Fig — (PDF) [file pcbi.1005192.s011.pdf]
