# Supplementary material for: Malaria Elimination Campaigns in the Lake Kariba Region of Zambia: A Spatial Dynamical Model
Source: PLoS Comput Biol. 2016 Nov 23;12(11):e1005192. doi: 10.1371/journal.pcbi.1005192 (PMC5120780; doi:10.1371/journal.pcbi.1005192)
Supplement: S10 Fig — Surveillance data was available for 8 out of 12 HFCAs. Dot size in scatter plot indicates relative cluster population. Line is y = x. To mirror interventions conducted in the area, simulations included ramp-up in case management, ITN mass distribution in July 2014, and a rudimentary reactive case detection where a random 10 individuals in a cluster are given AL when someone in the cluster receives treatment for clinical malaria. (PDF) [file pcbi.1005192.s012.pdf]

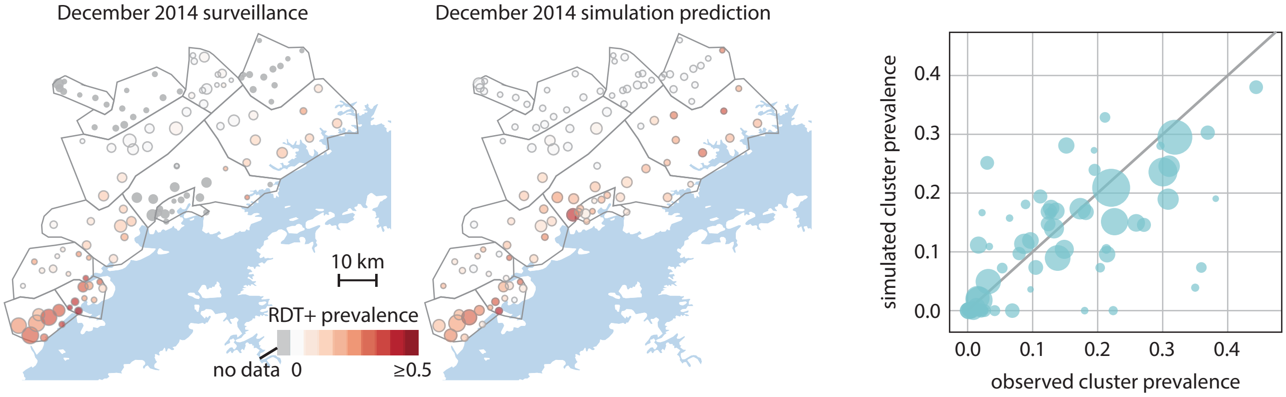

Figure S10. Out-of-sample prediction of December 2014 prevalence by RDT with the full spatial simulation. Surveillance data was available for 8 out of 12 HFCAs. Dot size in scatter plot indicates relative cluster population. Line is y = x. To mirror interventions conducted in the area, simulations included ramp-up in case management, ITN mass distribution in July 2014, and a rudimentary reactive case detection where a random 10 individuals in a cluster are given AL when someone in the cluster receives treatment for clinical malaria.
