# Supplementary material for: Malaria Elimination Campaigns in the Lake Kariba Region of Zambia: A Spatial Dynamical Model
Source: PLoS Comput Biol. 2016 Nov 23;12(11):e1005192. doi: 10.1371/journal.pcbi.1005192 (PMC5120780; doi:10.1371/journal.pcbi.1005192)
Supplement: S11 Fig — (A) ITN usage rates by age in 2015 and 2020 after ramp-up and aggressive distributions shown in S11C Fig and S11E Fig. (B) Usage rates of ITNs newer than 3 years old under the “maintain current coverage” scenario shown in S3B Fig. The spikes are due to the 1080-day interim between net distributions. (C, D) Usage rates of ITNs under the “ramp-up” scenario: (C) usage of any net and (D) usage of nets newer than 3 years old. (E, F) Usage rates of ITNs under the “aggressive” scenario: (E) usage of any net and (F) usage of nets newer than 3 years old. (PDF) [file pcbi.1005192.s013.pdf]

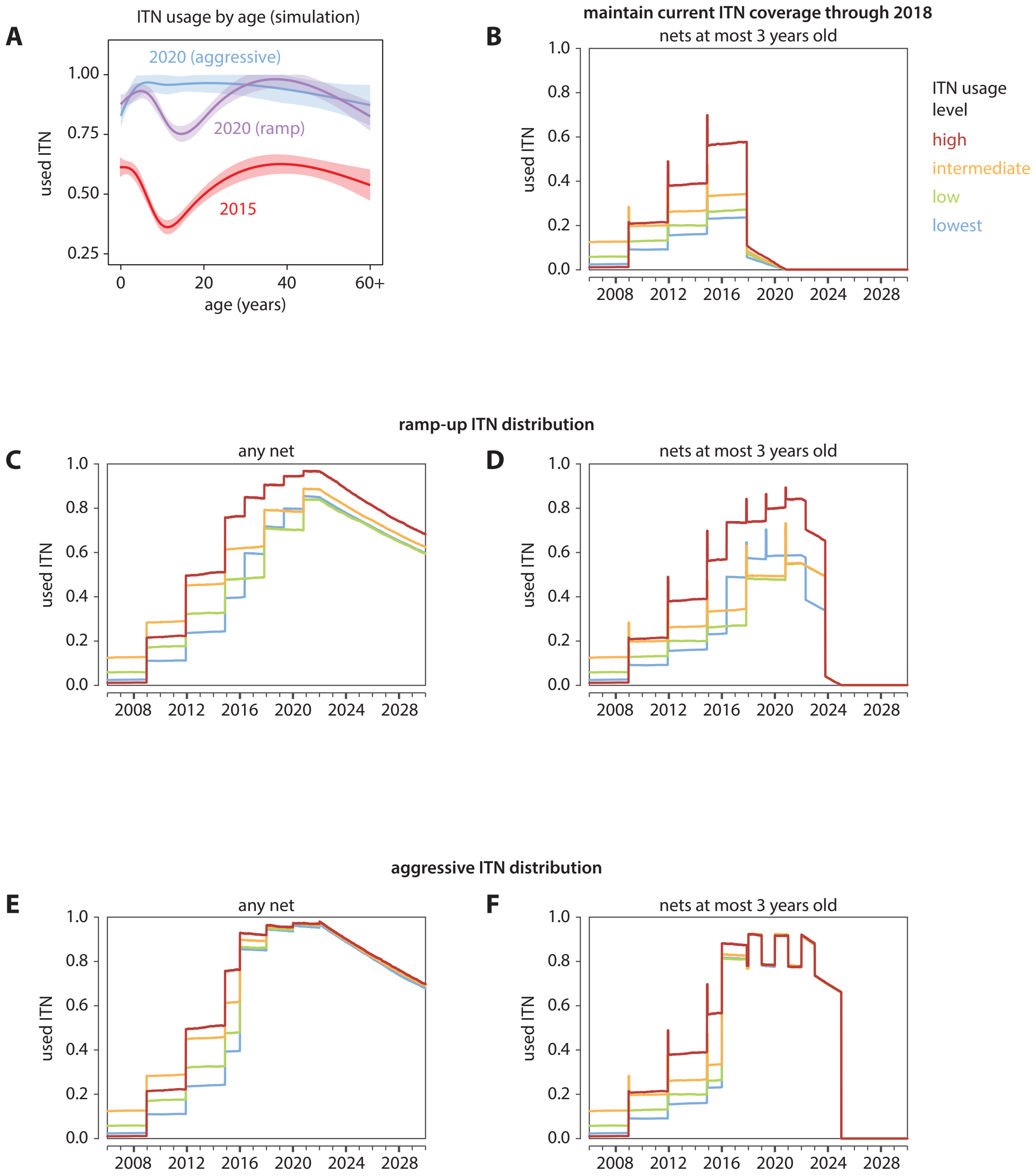

Figure S11. ITN ramp-up trajectories used in post-2015 intervention scenarios.
(A) ITN usage rates by age in 2015 and 2020 after ramp-up and aggressive distributions shown in Fig S11C and Fig S11E.
(B) Usage rates of ITNs newer than 3 years old under the "maintain current coverage" scenario shown in Fig S3B. The spikes are due to the 1080-day interim between net distributions.
(C, D) Usage rates of ITNs under the "ramp-up" scenario: (C) usage of any net and (D) usage of nets newer than 3 years old.
(E, F) Usage rates of ITNs under the "aggressive" scenario : (E) usage of any net and (F) usage of nets newer than 3 years old.
