# Supplementary material for: Malaria Elimination Campaigns in the Lake Kariba Region of Zambia: A Spatial Dynamical Model
Source: PLoS Comput Biol. 2016 Nov 23;12(11):e1005192. doi: 10.1371/journal.pcbi.1005192 (PMC5120780; doi:10.1371/journal.pcbi.1005192)
Supplement: S12 Fig — Single simulation of ramp-up in case management, aggressive ITN distribution, and 5 years of post-2015 MDA at historical coverage levels (S6 Fig) in all HFCAs. (PDF) [file pcbi.1005192.s014.pdf]

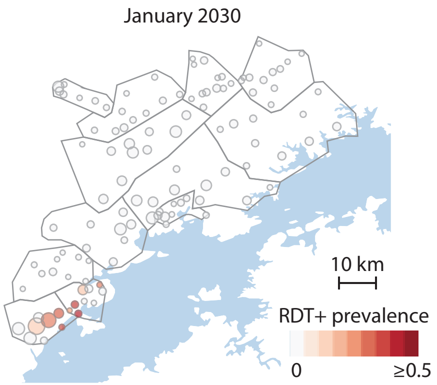

Figure S12. When elimination fails, transmission is re-established in high-population, high-transmission clusters. Single simulation of ramp-up in case management, aggressive ITN distribution, and 5 years of post-2015 MDA at historical coverage levels (Fig S6) in all HFCAs.
