# Supplementary material for: Malaria Elimination Campaigns in the Lake Kariba Region of Zambia: A Spatial Dynamical Model
Source: PLoS Comput Biol. 2016 Nov 23;12(11):e1005192. doi: 10.1371/journal.pcbi.1005192 (PMC5120780; doi:10.1371/journal.pcbi.1005192)
Supplement: S13 Fig — (A) Representative single simulation of scenario 13, where repeated importation of infections into the region results in reestablishment of transmission after vector control and MDAs cease in 2022. Prior to 2030, nearly all transmission is still localized to Sinamalima HFCA even though importations are also occurring in Munyumbwe and Gwembe HFCAs. (B) Maintaining vector control in areas with imported infections prevents reestablishment of transmission. In the yellow scenario, annual IRS campaigns with long-lasting insecticide (initial efficacy 0.6, half-life 9 months) at 75% coverage are distributed in all Sinamalima and Munyumbwe clusters between December 2022 and December 2029. (PDF) [file pcbi.1005192.s015.pdf]

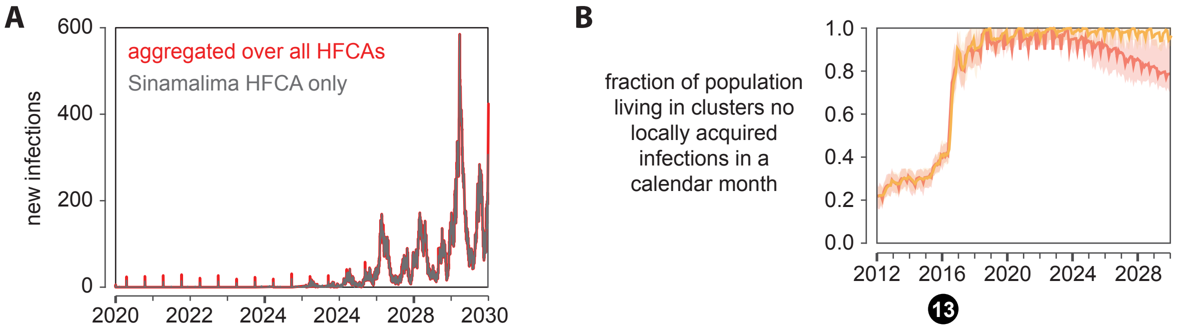

**post-2015 MDA**	all HFCAs, 2016-20	all HFCAs, 2016-20
**case management**	ramp	ramp
**ITN usage**	aggressive	aggressive
**migration rate**	low	low
**importation**	100 infections/year	100 infections/year
**vector control 2022-2030**	none	annual IRS in Munyumbwe and Sinamalima
**elimination?**	0/100	1/100

Figure S13. Impact of importations with and without routine vector control interventions.
(A) Representative single simulation of scenario 13, where repeated importation of infections into the region results in reestablishment of transmission after vector control and MDAs cease in 2022. Prior to 2030, nearly all transmission is still localized to Sinamalima HFCA even though importations are also occurring in Munyumbwe and Gwembe HFCAs.
(B) Maintaining vector control in areas with imported infections prevents reestablishment of transmission. In the yellow scenario, annual IRS campaigns with long-lasting insecticide (initial efficacy 0.6, half-life 9 months) at 75% coverage are distributed in all Sinamalima and Munyumbwe clusters between December 2022 and December 2029.
