# Supplementary material for: Malaria Elimination Campaigns in the Lake Kariba Region of Zambia: A Spatial Dynamical Model
Source: PLoS Comput Biol. 2016 Nov 23;12(11):e1005192. doi: 10.1371/journal.pcbi.1005192 (PMC5120780; doi:10.1371/journal.pcbi.1005192)
Supplement: S14 Fig — In these simulations, all individuals receiving DP as part of an MDA take only the first dose of the three-dose treatment regimen. (PDF) [file pcbi.1005192.s016.pdf]

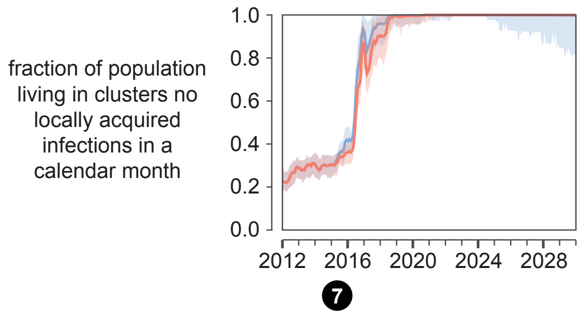

**post-2015 MDA**	all HFCAs, 2016-20	all HFCAs, 2016-20
**case management**	ramp	ramp
**ITN usage**	aggressive	aggressive
**migration rate**	low	low
**MDA compliance**	**high**	**low**
**importation**	none	none
**elimination?**	99/100	100/100

Figure S13. Elimination is equally likely under extremely poor compliance with MDA treatment when ITN coverage is aggressively increased. In these simulations, all individuals receiving DP as part of an MDA take only the first dose of the three-dose treatment regimen.
